# Lake-depth related pattern of genetic and morphological diatom diversity in boreal Lake Bolshoe Toko, Eastern Siberia

**DOI:** 10.1371/journal.pone.0230284

**Published:** 2020-04-15

**Authors:** Kathleen R. Stoof-Leichsenring, Katharina Dulias, Boris K. Biskaborn, Luidmila A. Pestryakova, Ulrike Herzschuh

**Affiliations:** 1 Polar Terrestrial Environmental Systems, Alfred Wegener Institute Helmholtz Centre for Polar and Marine Research, Potsdam, Germany; 2 Department of Geography and Biology, North-Eastern Federal University of Yakutsk, Yakutsk, Russia; 3 Institute of Environmental Science and Geography, University of Potsdam, Potsdam, Germany; 4 Institute of Biology and Biochemistry, University of Potsdam, Potsdam, Germany; University of Nottingham, UNITED KINGDOM

## Abstract

Large, old and heterogenous lake systems are valuable sources of biodiversity. The analysis of current spatial variability within such lakes increases our understanding of the origin and establishment of biodiversity. The environmental sensitivity and the high taxonomic richness of diatoms make them ideal organisms to investigate intra-lake variability. We investigated modern intra-lake diatom diversity in the large and old sub-arctic Lake Bolshoe Toko in Siberia. Our study uses diatom-specific metabarcoding, applying a short rbcL marker combined with next-generation sequencing and morphological identification to analyse the diatom diversity in modern sediment samples of 17 intra-lake sites. We analysed abundance-based compositional taxonomic diversity and generic phylogenetic diversity to investigate the relationship of diatom diversity changes with water depth. The two approaches show differences in taxonomic identification and alpha diversity, revealing a generally higher diversity with the genetic approach. With respect to beta diversity and ordination analyses, both approaches result in similar patterns. Water depth or related lake environmental conditions are significant factors influencing intra-lake diatom patterns, showing many significant negative correlations between alpha and beta diversity and water depth. Further, one near-shore and two lagoon lake sites characterized by low (0-10m) and medium (10-30m) water depth are unusual with unique taxonomic compositions. At deeper (>30m) water sites we identified strongest phylogenetic clustering in *Aulacoseira*, but generally much less in *Staurosira*, which supports that water depth is a strong environmental filter on the *Aulacoseira* communities. Our study demonstrates the utility of combining analyses of genetic and morphological as well as phylogenetic diversity to decipher compositional and generic phylogenetic patterns, which are relevant in understanding intra-lake heterogeneity as a source of biodiversity in the sub-arctic glacial Lake Bolshoe Toko.

## Introduction

Large lake systems serve as biodiversity hotspots and are famous for their endemic flora and fauna. The origin of new biodiversity is generally attributed to the lake’s condition, which provides heterogeneous habitats in which organisms have diversified over a long time under generally stable and undisturbed conditions and are, at the same time, highly susceptible to environmental perturbations [1–3]. Relevant lake conditions in the context of biodiversity formation are lake size, ecosystem age and habitat variability like water depth gradient or differences in the catchment area [2]. Diatoms are minute, quickly reproducing eukaryotic algae, which are ecologically extremely sensitive and diverse [4]. They are a predominant component of lake sediments and are known to be suitable bioindicator proxies used in spatial and temporal lake environmental reconstructions [5–7]. Moreover, studies on large old lake systems have uncovered several endemic diatom taxa [8–11], which make them suitable organisms to investigate their community patterns in the light of taxa differentiation and formation of biodiversity. It is known that differences in diatom communities in various intra-lake settings have been attributed to strong variations in lake depth, light exposure, ice cover dynamics, habitat variability and lake chemistry including pH, salinity, phosphorous and nitrogen concentrations, as well as to catchment-related differences [11–16], which suggests that these parameters especially influence the biodiversity of such systems. Traditionally, diatoms are identified by microscopy, which is very time-consuming and needs an in-depth taxonomic knowledge. In addition, the detection of diatom diversity by light microscopy is limited as ecological differences are not necessarily mirrored by differences in morphological traits. Especially regarding within-species/genus variations, whereas several genetic approaches addressing diatom diversity on lower taxonomic levels have uncovered cryptic and hidden diversity in similar morphotypes [17–19]. With the advent of the metabarcoding approach combined with advancing high-throughput next-generation sequencing techniques, diatom compositions have been retrieved with various genetic markers and from different environments [20–24]. Especially differences in intra/inter-specific variations have been detected in sedimentary DNA (sedDNA) from surface- and core-sediment samples by applying a short, but specific, chloroplast rbcL metabarcode [25,26]. This ability for in-depth taxonomic identification in some diatom groups offers a great advantage for the analysis of minor differences between lakes, but also within-lake variability of ecologically indicative minute diatoms. In general, genetic and morphological investigations of diatoms from water or sediment samples show a good overlap between the overall taxa composition, although genetic studies show stronger differences in the relative abundance of sequence reads per sample and indicate higher diversity as well as assigning taxa to a higher taxonomic level depending on the completeness of the reference database [22,24,27]. Besides retrieving taxonomic community compositions by metabarcoding, phylogenetically informative genetic markers also provide an estimation of the phylogenetic diversity [28], which is limited in short barcodes but can be sufficient to display phylogenetic patterns within genera [29]. In general, phylogenetic diversity analyses allow the assessment of whether communities are phylogenetically clustered, which is mostly caused by environmental filtering or if they are phylogenetically over dispersed, which can be a result of biotic interactions [30,31]. The phylogenetic signals can identify relatedness patterns between lineages in a community, which can indicate rates of diversification and help to elucidate hotspots of biodiversity and their putative environmental drivers [32].

The basin structure of the large and deep permafrost lake “Lake Bolshoe Toko” likely facilitates intra-lake variability with large variations in lake depth, making Lake Bolshoe Toko an interesting study area to explore different facets of diatom biodiversity with respect to varying water depth. With our study we aim to evaluate (i) if morphologically and genetically retrieved intra-lake diatom composition are similar, (ii) if retrieved diatom composition is in part explained by lake water depth and (iii) how phylogenetic signals in diatom genera relate to lake water-depth variations. We will evaluate the effect of lake water-depth variations on the taxonomic and phylogenetic diatom diversity to elucidate modern biodiversity patterns in the large subarctic Lake Bolshoe Toko.

## Materials and methods

### Study site

**Fig 1:**
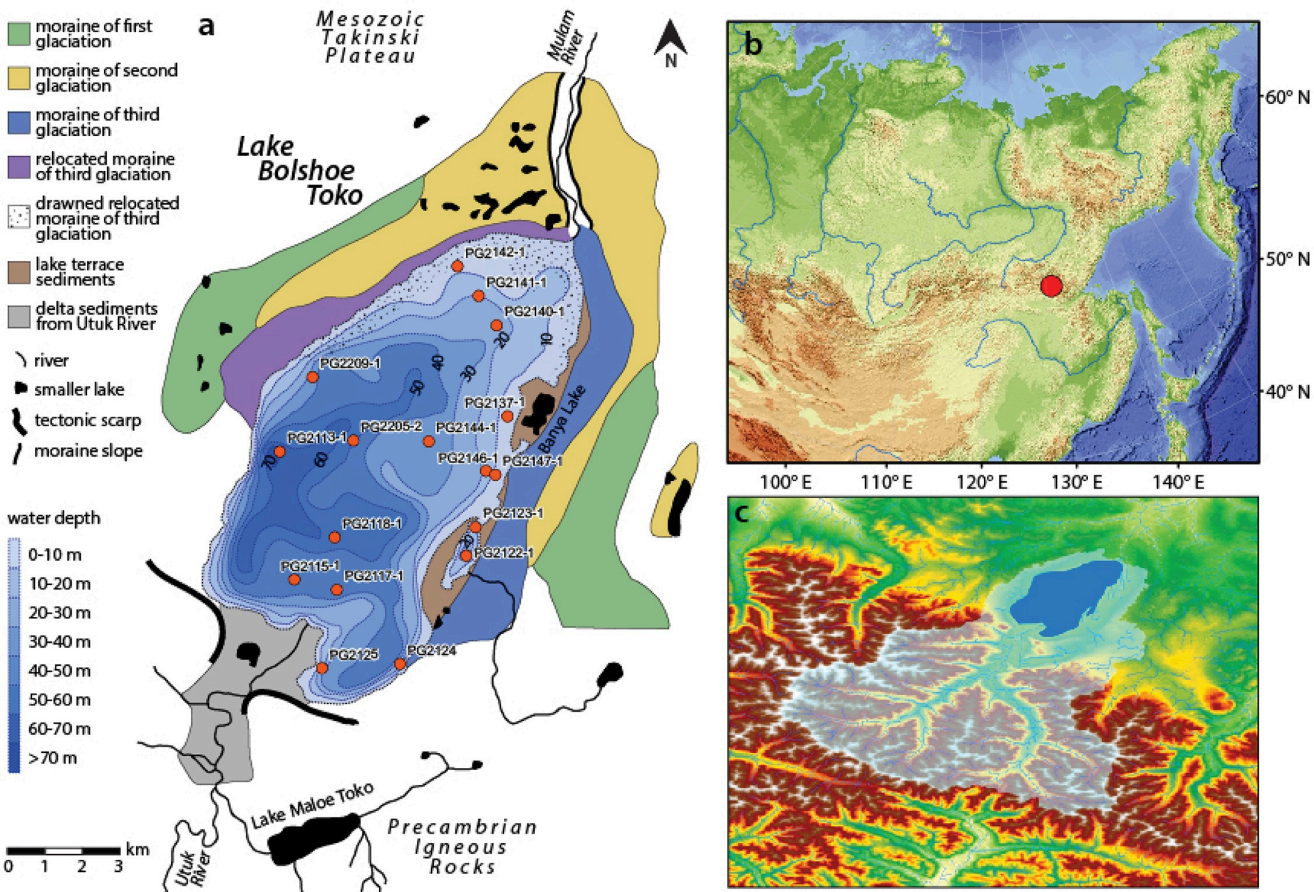
Lake bolshoe toko study site. **a** Geological map, bathymetry and moraines. Map compiled using data from [33] and [34]. **b** Overview map of Siberia. Background image reproduced from the GEBCO World Map 2014 (www.gebco.net). **c** Catchment area around Lake Bolshoe Toko delineated from the ASTER GDEM V2 model between the latitudes N54° and N56° and longitudes E130° to E131°.

Lake Bolshoe Toko (56°15’N, 130°30’E, 903 m.a.s.l., length: 15.4 km, width: 7.4 km, maximum water depth: 80 m, surface area: 82.6 km^2^) is located in southeastern Yakutia, Sakha Republic, Russia (Fig 1).

Three moraines of different, but unknown age, border the northern margin of the lake. The northeastern part of the lake basin is shallower (<30 m) compared to the southwestern part of the lake that can reach up to 80 m water depth. The Utuk River runs through the smaller but deeper Lake Maloe Toko supplying Bolshoe Toko with water from the southern catchment in the Stanovoy mountain range. The lake catchment is located in the East Siberian continental temperate climate zone with a predominant westerly wind system [35]. According to data from the meteorological station Toko, located ca. 10 km northeast of Lake Bolshoe Toko, mean annual air temperature is 11.2°C, ranging from -65°C in January to +34°C in July. Annual precipitation is between 276–579 mm. Satellite data show that the spring ice break was on 12th June for year 2017 and that, as in other years, the ice lasted longer in the northeast area of the lake. The influence of cold water from the Stanovoy mountain range causes slightly lower summer temperatures and high ice volume during winter [33]. Permafrost is reportedly discontinuous in some local areas, experiencing strong large-scale warming [36]. Unfrozen zones (taliks) beneath deep lakes such as Lake Bolshoe Toko are believed to reach the permafrost base [37]. Recently, a small lagoon on the eastern shore of the lake is gradually becoming separated from the main basin, presumably due to increasing temperature and lake level decrease [38]. According to [39] Lake Bolshoe Toko, as compared to all other investigated lakes in the study, is the deepest lake in Yakutia. There is evidence from past shorelines that its basin structure resulted from three different glacial advances. Unpublished radiocarbon dates of a 3.8 m long sediment core, taken during the expedition mentioned above, reach back to max. 44,000 years BP, but given the short length of the core a greater age for the Lake Bolshoe Toko is expected.

### Sample retrieval

The Russian-German expedition “Yakutia 2013” was conducted by the Alfred Wegener Institute Helmholtz Centre for Polar and Marine Research and the North Eastern Federal University Yakutsk from March 19^th^ to April 14^th^ in 2013. To reach the sediment through the ice cover of around 1 m, we drilled ice holes using a Jiffey ice auger. Short sediment cores of up to ca. 40 cm length were retrieved at multiple sites within the lake, using a UWITEC gravity corer [38]. Sediment cores were immediately subsampled after retrieval in the field for DNA analyses. Each DNA sample was taken from the middle of each 1–2 cm sediment slice in the respective sediment cores, without touching the outer part of the slice. The DNA samples were taken with a sterile spatula and were collected in 8 ml sterile screw tubes (Sarstedt) containing Queens Tissue buffer [40]. Samples for microscopic diatom investigations were obtained from the same core always using the 0–0.5 or 0–1.0 cm of the core record, according to in-field subsampling strategies. DNA and microscopic samples were kept cold at 4°C and dark until they were analyzed in the genetic and sedimentological laboratories at the Alfred Wegener Institute in Potsdam, Germany. For this study, we used 17 intra-lake sites from which both DNA and morphological analysis are available (Fig 1 and S1 Table). All permits to work on lake Bolshoe Toko was obtained by the Russian partners of the Russian-German expedition Yakutia 2013 managed by North-Eastern Federal University of Yakutsk (NEFU). Field site access was approved by the Ministry of Ecology, Nature Management and Forestry of the Republic of Sakha.

### Genetic diatom approach

DNA extractions of 21 sediment samples were performed using the PowerSoil® DNA Isolation Kit (MoBio Laboratories, California). Samples were processed in a dedicated isolation laboratory using a dedicated UV-hood for extraction of environmental DNA and within each extraction a blank control was included. We mainly followed the manufacturer’s protocol, but used the FastPrep 24^TM^ instrument (MP Biomedicals) for sediment lysis (full speed for 50 sec) and eluted the DNA in 50 μl C6 buffer. We amplified a short metabarcode, a fragment of the ribulose-1,5-bisphosphate carboxylase ⁄ oxygenase large subunit (rbcL), specified to gain diatom diversity from sedimentary samples (25,26) by using PCR with barcoded primers. Preparation of PCR samples for DNA sequencing are described in (24). Based on the metafast protocol, the pool of PCR samples was sequenced with parallel high-throughput paired-end (2x125 bp) amplicon sequencing on the Illumina HiSeq 2000 platform (Illumina Inc.) using an external sequencing service (Fasteris SA, Switzerland). The obtained sequence data were analyzed using OBITools, basically following the steps described in [41]. Taxonomic assignment with the function *ecotag* was performed using a database created with ena release128. Details of the bioinformatical analyses of the data are given in [24]. After using the OBITools pipeline, the final results were entered into a spreadsheet and further filtered manually. In a first step, we only included sequence types that had the exact length of the rbcL metabarcode of 76 bp and were at least assigned to the phylum Bacillariophyta. All sequences that did not have the exact length of 76 bp and assigned as non-diatom sequences were removed. Furthermore, sequence counts found in the extraction blanks or negative template controls (NTCs) (in total less than 0.1% of all sequences) were subtracted from the relevant samples belonging to each extraction and/or PCR batch. Sequences that occurred less than three times independently and with a relative abundance lower than 0.01% were also excluded from the data. Thus, the final dataset includes only a selection of 17 out of 21 samples because of missing morphological data and includes only reads that are at least identified to the diatom family.

### Morphological diatom approach

Seventeen surface sediment samples from Lake Bolshoe Toko were prepared for diatom analysis following standard procedure described in [5]. Organic matter was removed using hydrogen peroxide and slides were prepared on a hot plate using Naphrax mounting medium. For the identification of diatoms several diatom flora were used including [42], [43], [44] and [45]. For *Pliocaenicus* we used [46] specifically for Bolshoe Toko. A minimum of 300 (and up to 450) diatom valves were counted in each sample. Identification of diatom valves was performed using a Zeiss AXIO Scope.A1 light microscope with a Plan-Apochromat 100×/1.4 Oil Ph3 lens at 1000x magnification.

### Statistical analyses

#### Diversity metrics

Strong differences in total sequence counts of the DNA dataset require rarefaction of the genetic raw data prior to further statistical analyses. Therefore, DNA sequence counts were rarefied to the minimum sample count (total count = 4709) and resampled (100 times) to create random datasets. Mean values of the 100-resampled datasets are used to create the final genetic dataset. For rarefaction analyses we use the R script of Stefan Kruse (https://github.com/StefanKruse/R_Rarefaction). The morphological dataset is not rarefied, because there are very similar total counts per sample (mean 361±34.019). For both datasets we calculated richness and hill numbers as a measure of taxonomic alpha diversity in the intra-lake sampling localities using the R package “iNEXT” [47]. The hill number to the order of q = 2 estimates the number of effective species per sample, by calculating the reciprocal of the Simpson diversity index. Taxa diversity, sorted according water depth of sampling localities, for both datasets is visualized in bar plots and the main diatom groups (araphid, raphid, centric) summarized using Tilia version 2.1.1 (copyright 1991–2016 Eric C. Grimm). We calculated beta diversity of both datasets using the function *beta*.*div* in the R package “adespatial” [48], which estimates the total beta diversity (BD_Total_) from the total variance in the community data and decomposes the BD_Total_ into species contribution to beta diversity (SCBD) and locality contribution to beta diversity (LCBD). Prior to the calculations of beta diversity both datasets were transformed using Hellinger dissimilarity and the estimated *p*-values of LCBD were corrected using the Holm method.

The morphological and the rarefied genetic datasets were Hellinger-transformed prior to applying ordination methods [49]. A principal component analysis (PCA) was performed for both datasets and PROCRUSTES rotation analysis [50] was applied to compare the resulting ordinations with regard to their shape similarity. Significance of similarity between the tested datasets is assessed by PROTEST [51]. The environmental variable water depth was log-transformed. Further, we used redundancy analysis (RDA) to investigate the relation between the water depth and diatom composition in both datasets. Ordination analyses were carried out and plotted with *ordiplot* using the “vegan” package [52], scatter and bar plots were created using the package “ggplot2” [53] and all computations were conducted with RStudio version 1.1.456 © 2009–2018 RStudio, Inc.

#### Phylogenetic diversity

The determination of alpha phylogenetic diversity is based on the mean phylogenetic distance (mpd) of generic communities in the single intra-lake localities, but generally displays phylogenetic divergence rather than phylogenetic richness within a sample locality [31]. We used rarefaction as described above to rarefy generic data sets of *Aulacoseira* (minimum sample count = 50) and *Staurosira* (minimum sample count = 45) prior to the calculation of mpd. The calculation of mpd was conducted by estimating the tree branch lengths from a maximum likelihood tree using the function *pml* (with the GTR substitution model) implemented in R package “phangorn” [54], which were calculated from sequence types detected at each sampling locality. Then we used R package “picante” [55] and coupled phylogenetic distances to the abundance of sequence types with the function *match*.*phylo*.*comm*. We calculated mpd by using the function *ses*.*mpd*. This function compares mpd values retrieved from random null communities and observed communities and estimates significant deviations in observed (either based on abundance or presence/absence data) from random communities. This estimate (mpd.obs.z) can be converted to the net relatedness index (NRI) by multiplying by -1. Significant positive NRI values indicate a co-occurrence of closely related lineages indicating environmental filtering, whereas significant negative values presume the co-occurrence of distantly related lineages interpreted as exclusive biotic interactions [30]. Phylogenetic diversity relates to the average of mpd in a community, but displays phylogenetic divergence rather than phylogenetic richness within a sample locality [31]. We tested linear relationships between generic NRI values, richness and water depth. Linear regression analyses were performed using the *ggscatter* function implemented in the R package “ggpubr v.0.2.3” [56]. Plots and linear regression lines were produced using *ggplot* and *geom_smooth*(method = lm) function in R package “ggplot2” [53].

## Results

### Taxonomic intra-lake diatom composition and diversity

We retrieved genetic and morphological diatom compositions from 17 lake surface-sediment samples. The genetic data consist of 1,199,458 paired reads, whereof 1,126,014 (93.9%) have the exact length of 76 bp and are at least assigned to the phylum Bacillariophyta (in total 280 unique sequence types). After reducing the dataset to at least sequence types assigned to family, to enable comparison with morphological results, the dataset consists of 955,926 (79.7% of the total raw data) reads and the selection of 17 sampling sites reduces the data to 853,446 sequence reads comprising 212 unique diatom sequences types, whereof 40% could be identified to genus and 45% to species level. All of the sequence types show an identity of at least 90% to the reference sequences (S2 Table). On average 132 different sequences types are identified per sample location. Despite equimolar pooling of PCR products for multiplex sequencing [24], the total number of sequence reads varies substantially among samples: between 4709 and 218,963 with a median of 30,943. The total morphological dataset includes 109 species identified from 61,636 total counts, the number of counts per sample ranges between 304 and 450 and the average number of species identified per sample location is 34.

**Fig 2:**
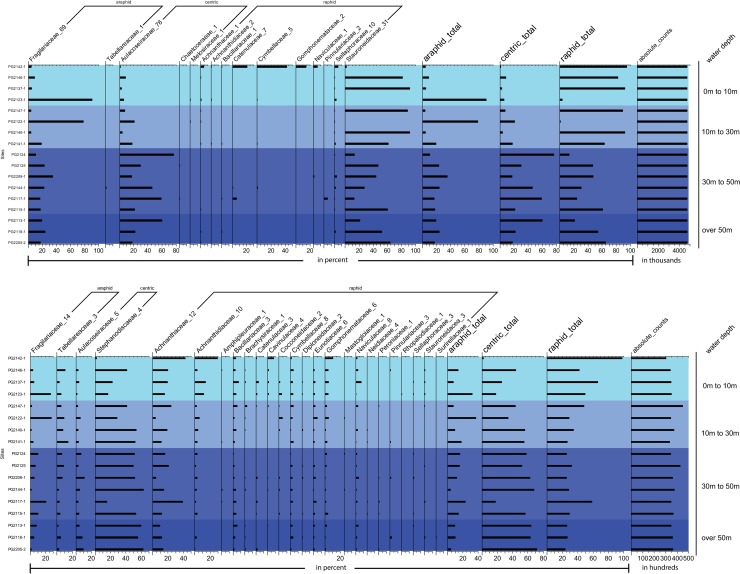
Diatom relative abundance. Percentages of diatoms retrieved by the genetic (a) and morphological (b) approach of surface samples from Lake Bolshoe Toko ordered by water depth. Numbers following the family names are the number of retrieved distinct sequence or morphological diatom types identified as belonging to the diatom family. Family assigned sequences and counts are summed up in three groups–araphid, raphid and centric diatoms. The genetic dataset has been rarefied to account for large differences in sequence reads per site. Morphological data are not rarefied as the number of absolute counts does not differ significantly.

The taxonomic assignment of the sequence types identifies Aulacoseiraceae, Fragilariaceae and Stauroneidaceae as the most frequently recorded diatom families (Fig 2A), whereof sequence types assigned to the genus *Aulacoseira* are the most abundant and diverse, followed by sequence types assigned to *Staurosira ellipta* and *Stauroneis constricta* (S2 Table).

The morphological approach identified mostly the families Stephanodiscaceae, Achnanthaceae, Achnanthidiaceae, Fragilariaceae, Tabellariaceae and Aulacoseiraceae (Fig 2B), whereof the species *Pliocaenicus bolshetokoensis* [46], *Achnanthidium minutissimum* and *Cyclotella comensis* are most abundant (S2 Table). The highest diversity is detected in the families Fragilariaceae, Aulacoseiraceae and Stephanodiscaceae (Fig 2B).

The diversity metrics obtained from the genetic and morphological datasets from surface-sediment samples of Lake Bolshoe Toko have contrary results for alpha diversity. The average richness per sample from the genetic approach is 97 and the effective number of species is, on average, 5. In particular, the samples PG2137-1 (0-10m), PG2140-1 (10-30m) and PG2142-1 (0-10m) show a very low diversity (Table 1). The average richness per sample from the morphological data is 34, the mean effective number of species is 9.4 and samples PG2142-1 (0-10m), PG2205-2 (>50m) and PG2124 (30-50m) show the lowest diversity. Although both datasets indicate differences in the alpha diversity, they collectively identify sample PG2142-1 (0-10m) as the sample with the poorest richness, and also the most different site in terms of diatom community. The diatom composition at this site is composed of diatoms from the families Achnanthidiaceae, Catenulaceae, Cymbellaceae, Gomphonemataceae, Naviculaceae and Sellaphoraceae as revealed by the genetic approach and high frequencies of diatoms from the families Achnanthidiaceae, Cavinulaceae, Gomphonemataceae and Naviculaceae as identified morphologically (Fig 2).

**Table 1:** Lake parameters. Lake sampling sites, water depth and result of the diversity analyses of genetic and morphological data.

		Genetic data							Morphological Data					
lake sites	water depth (m)	total counts	rarefied counts	richness	Effective number of species (Hill2)	LCBD	*p*LCBD	adj *p*LCBD	total counts	richness	Effective number of species (Hill2)	LCBD	*p*LCBD	adj *p*LCBD
**PG2142-1**	0	4709	4709	55	5.22	0.192	0.001***	0.017*	304	23	8.98	0.186	0.001***	0.017*
**PG2146-1**	5	11650	4709	100	1.59	0.024	1	1	341	57	11.34	0.049	0.645	1
**PG2137-1**	5.8	42938	4709	67	1.24	0.042	0.661	1	348	44	17.52	0.057	0.494	1
**PG2123-1**	6.1	29891	4709	92	10.58	0.13	0.036**	0.112	350	46	13.84	0.119	0.009**	0.105
**PG2147-1**	11	45014	4709	86	1.34	0.032	0.939	1	450	38	10.08	0.033	0.966	1
**PG2122-1**	18.3	33303	4709	103	10.57	0.121	0.059^.^	0.33	344	31	8.35	0.136	0.001***	0.080^.^
**PG2140-1**	25	113228	4709	68	1.24	0.039	0.747	1	372	33	8.61	0.03	0.989	1
**PG2141-1**	27	30943	4709	140	2.86	0.017	1	1	334	43	9.2	0.052	0.569	1
**PG2124**	30	22548	4709	95	4.73	0.072	0.233	1	343	26	7.66	0.035	0.939	1
**PG2125**	30	63588	4709	142	6.17	0.027	0.991	1	428	28	8.09	0.03	0.98	1
**PG2209-1**	31.2	58606	4709	75	4.15	0.038	0.75	1	360	37	10.63	0.044	0.759	1
**PG2144-1**	36.8	14034	4709	88	7.64	0.05	0.451	1	377	32	7.44	0.037	0.911	1
**PG2117-1**	36.9	218963	4709	120	11.3	0.103	0.147	1	347	32	5.55	0.069	0.258	1
**PG2115-1**	45.5	19675	4709	118	3	0.021	1	1	367	30	9.38	0.029	0.994	1
**PG2113-1**	62	91930	4709	75	7.12	0.057	0.328	1	347	31	8.12	0.03	0.986	1
**PG2118-1**	62.3	14932	4709	111	3.81	0.02	1	1	359	29	8.23	0.033	0.97	1
**PG2205-2**	68.3	26919	4709	116	2.61	0.016	1	1	365	25	7.48	0.03	0.994	1

LCBD–Local contribution to beta diversity, adj *p*LCBD–adjusted *p* values for LCBD. *p* values are coded in *** 0.001, **0.01, *0.05, ^●^ 0.

In contrast to the results of alpha diversity, the total beta diversity (BD_Total_) in the genetic (0.38) and the morphological datasets (0.31) is very similar. The SCBD for the genetic dataset is on average 0.005 and the main contributors to total beta diversity are sequence types *Stauroneis* (Stu_28), *Aulacoseira* (Aul_71, Aul_53), Cymbellacaea (cym_4) and *Staurosira elliptica* (Sta_25). The average SCBD in the morphological dataset is 0.009: mainly *Cyclotella comensis* (Cyc_com), *Cyclotella cyclopuncta* complex (Cyc_cyc_complex), *Achnanthidium minutissimum* (Ach_min), *Fragilaria pinnata* (Fra_pin) and *Pliocaenicus bolshetokoensis* (Pli_bol) (S2 Table).

Both approaches reveal significant LCBD values for the two lagoon sites PG2122-1 (10-30m) and PG2123-1 (0-10m) and the near-shore site PG2142-1 (0-10m), indicating the ecological uniqueness of these lake localities compared to other lake sites. However, after Holm correction, only the sample PG2142-1 (0-10m) is significant, whereas the other localities have only weak statistical support (Table 1).

### Taxonomic intra-lake diatom diversity and its relation to lake water depth

**Fig 3:**
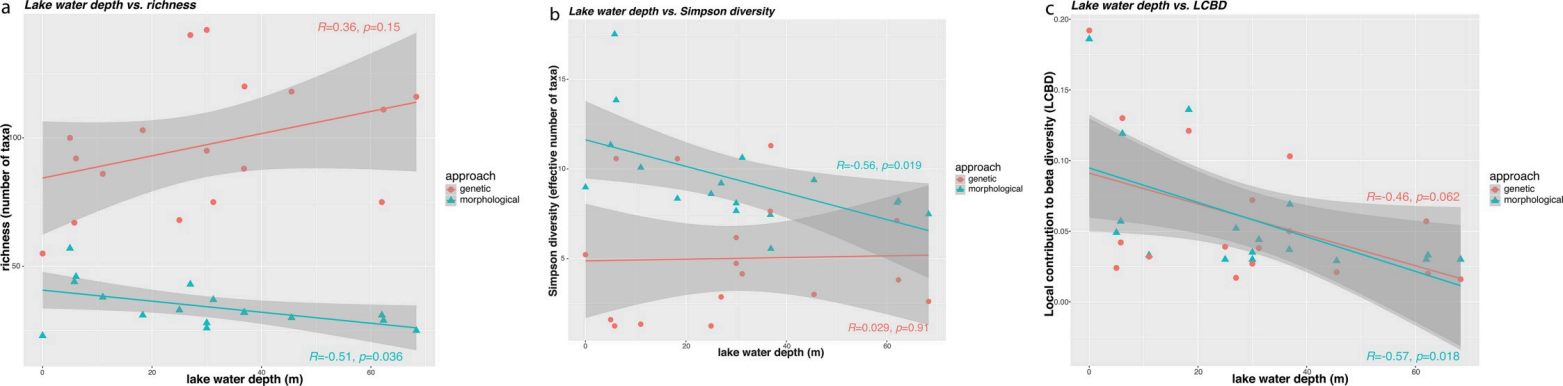
Diversity metrics. Relationship between diversity metrics obtained from the genetic (red) and morphological (blue) diatom data showing water depth with (a) richness (number of taxa), (b) effective number of species and (c) local contribution to beta diversity (LCBD).

The relationship between alpha diversity and water depth indicates a significantly negative correlation with morphology (R^2^ = 0.26, *p* = 0.036 for richness and R^2^ = 0.31, *p* = 0.019 for hill2). The genetic data show a very weak positive relationship with richness (R^2^ = 0.13, *p* = 0.15), but no significant relationship (R^2^ = 0.0008, *p* = 0.91) with water depth and effective number of species (hill2) diversity (Fig 3A and 3B). In samples having a water depth between 0-10m the morphological diatom community is most diverse (mean effective number of species 12.5) compared to deeper lake depths (mean effective number of species 8.3). Diverse raphid and araphid epiphytic/benthic diatoms are present in shallow waters (*Achnanthidium*, *Psammothidium*, *Fragilaria*, *Nitzschia*, *Eucocconeis*, *Reimeria*) but also planktonic centric diatoms occur at high numbers (*Plioceanicus*, *Cyclotella*). With water depth over 10m the diatom community shifts to a dominance of planktonic taxa (*Plioceanicus*, *Cyclotella*, *Aulacoseira*), but epiphytic taxa *Achnanthidium* and *Fragilaria* are also detected (S3 Table). The genetic data as well mirror a shift from pennate to centric diatoms at >10m water depth. The community in shallow lake sites <10m is dominated by *Stauroneis* (Stu_28), Cymbellaceae (cmy_4), *Staurosira elliptica* (Sta_25) and Gomphonemaceae (gom_1). Below 10m the community is composed of different Aulacoseiraceae types (Aul_71, 78, 70), *Stauroneis* (Stu_28) and *Staurosira* (Sta_25); with deeper water depth additional sequence types of *Aulacoseira* and *Staurosira/Fragilaria* accompany the community (Fig 2 and S4 Table). In contrast to alpha diversity, scatterplots comparing the relation between water depth and beta diversity indicate that both approaches show a significant negative correlation (genetic approach R^2^ = 0.21, *p* = 0.062; morphological approach R^2^ = 0.33, *p* = 0.018) indicating a decrease in taxa turnover with increasing water depth.

**Fig 4:**
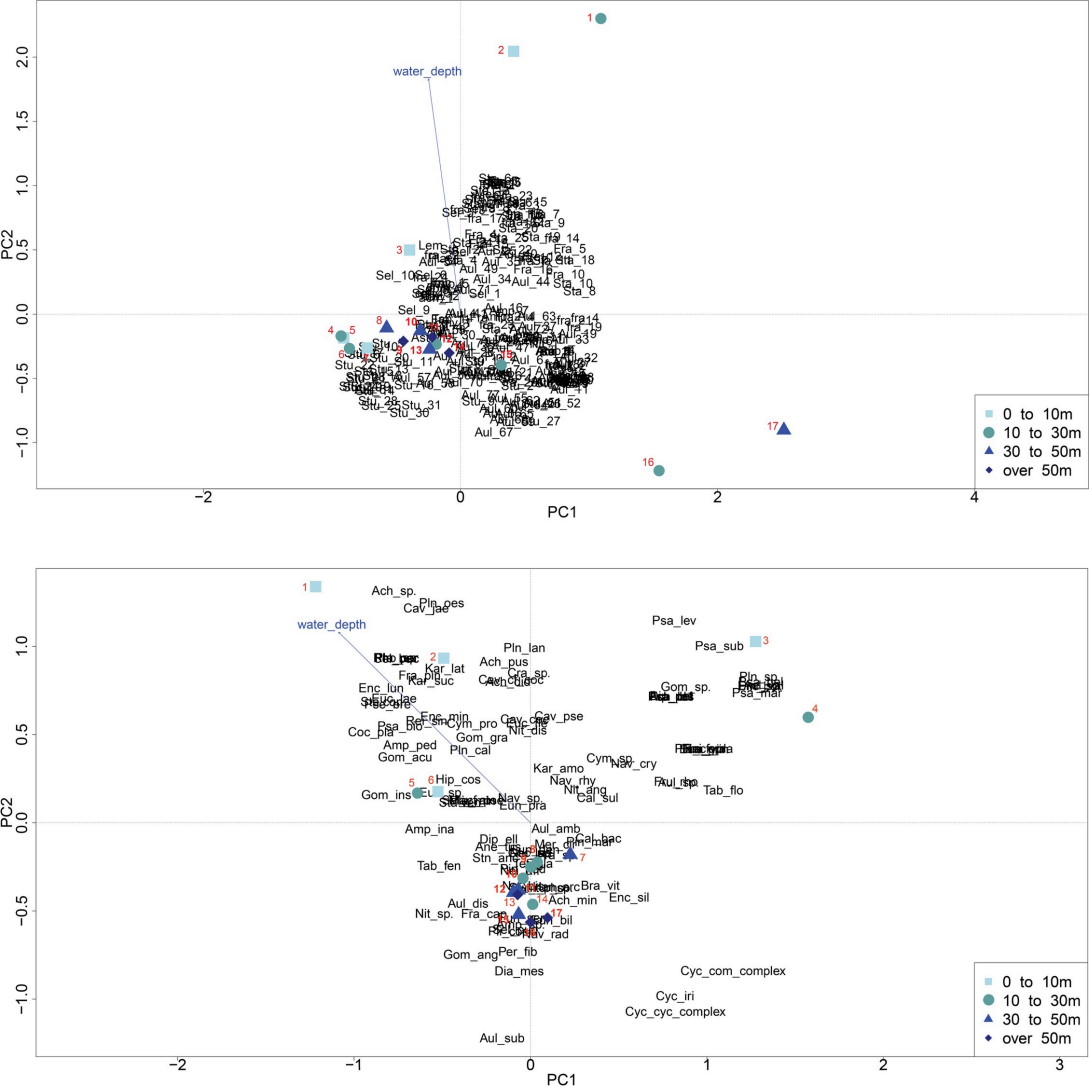
Principal component analysis (PCA). PCA of the diatom assemblies retrieved from the genetic approach (a) and the morphologically identified diatom taxa (b) with the water depth fitted on the ordination plot. Numbers in the genetic plot denote the following sites: 1 –PG2122, 2 –PG2123, 3 –PG2142, 4 –PG2140, 5 –PG2137, 6 –PG2147, 7 –PG2146, 8 –PG2209, 9 –PG2205, 10 –PG2144, 11 –PG2118, 12 –PG2141, 13 –PG2115, 14 –PG2113, 15 –PG2124, 16 –PG2125, 17 –PG2117. Numbers in the morphological plot denote the following sites: 1 –PG2123, 2 –PG2142, 3 –PG2146, 4 –PG2141, 5 –PG2122, 6 –PG2137, 7 –PG2144, 8 –PG2147, 9 –PG2140, 10 –PG2124, 11 –PG2209, 12 –PG2117, 13 –PG2113, 14 –PG2125, 15 –PG2115, 16 –PG2205, 17 –PG2118.

The principal component analysis (PCA) shows that 24.2% and 21.87% of the variance of the diatom assemblages of the rarefied genetic dataset are explained by the first and second principal components, respectively, resulting in a cumulative proportion of 46.07% (Fig 4A). For the morphological species dataset, PC1 and PC2 explain 15.81% and 14.23%, respectively, of the variance in the morphological diatom assemblage, with a cumulative proportion of 30.04% (Fig 4B). In the genetic dataset, sites PG2117-1 (30-50m), PG2122-1 (10-30m), PG2123-1 (0-10m) and PG2125 (30-50m) differ from the other samples based on the variance of water depth (explained by the second principal component), whereas PC1 accounts for their spread into the upper and lower right quadrants of the PCA. The PCA of the morphological data shows that sites PG2123-1, PG2142-1, PG2146-1 (all at 0-10m) and PG2141-1 (10-30m) differ from the other sites both based on the variance of the water depth (PC2) and the variance of PC1. To evaluate the similarity of ordinations obtained from the genetic and the morphological data we applied the PROCRUSTES analyses and PROTEST to test the significance (number of permutations = 999) between the two configurations, which revealed a correlation in a symmetric PROCRUSTES rotation (m12 = 0.75, r = 0.51, *p*-value = 0.018, RMSE = 0.21), indicating resemblance between both datasets (S1 Fig). The constrained variance of the water depth is calculated by RDA and explains 2.75% and 3.06% of the variance in the rarefied genetic and the morphological data sets, respectively.

### Taxonomic and phylogenetic diversity and its relation to lake depth in diatom genera

Phylogenetic diversity analyses were performed on two dominant diatom genera *Aulacoseira* comprising 78 intra-generic lineages and *Staurosira* with 25 intra-generic lineages (S5 Table). Prior to phylogenetic analyses we tested the correlation between water depth and richness for both genera data sets (S2 Fig). For *Aulacoseira* our results indicate a significant positive correlation between water depth and richness (R^2^ = 0.27, *p* = 0.033), whereas there was no correlation for *Staurosira* (R^2^ = 0.016, *p* = 0.800).

The phylogenetic diversity is calculated based on the comparison of mpd in observed and random communities resulting in the net relatedness index (NRI). Because difference in richness among the analyzed sites can affect NRI values, we checked possible correlations between richness with NRI values (S2 Fig). For *Aulacoseira* our results reveal no relationship between richness and NRI (R^2^ = 0.017, *p* = 0.61). For *Staurosira* we detected a significant negative correlation between richness and NRI (R^2^ = 0.28, *p* = 0.034).

**Fig 5:**
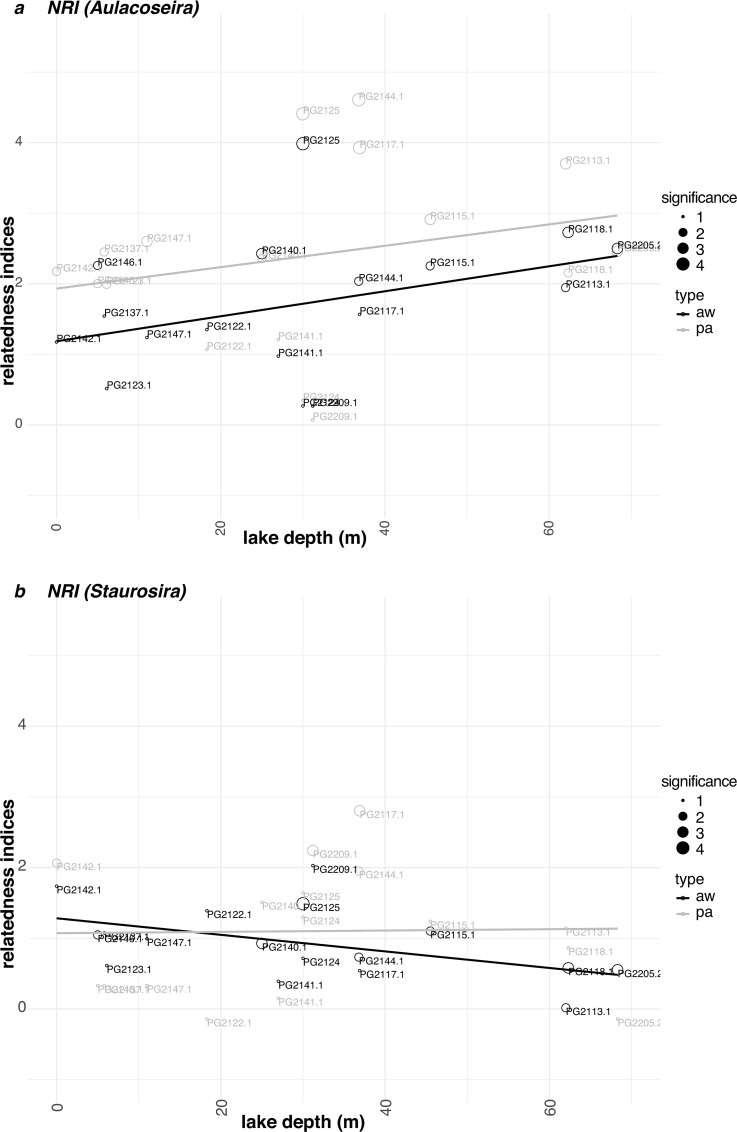
Relationships between diversity measures and water depth. Relationship between richness, lake water depth (m) and net relatedness index (NRI; = standardized effect size of mean phylogenetic distance (mpd) of observed vs. null communities) of sequence types of the genus *Aulacoseira* (a) and *Staurosira* (b) for the 17 intra-lake sampling sites. Significant deviations from null communities are indicated by the circle size (1 –p>0.05; 2 –p≤0.05; 3 –p≤0.01; 4 –p≤0.001).

*Aulacoseira* communities for most of the intra-lake samples present a significant positive NRI (using presence/absence data as input), which indicates a co-occurrence of more closely-related sequence types in one locality compared to null communities (Fig 5A). Although there is no significant linear relationship between NRI and water depth (R^2^ = 0.063, *p* = 0.34), there is the tendency that with increasing water depth NRI values become more significant and interestingly, values show a peak at localities (PG-2125, PG2144.1, PG2117.1) in water depths between 37–30 meters (Fig 5A). The NRI results obtained from abundance-weighted data input show less significant values than estimated by presence/absence data, but generally follow the same trend. For *Staurosira* communities, only a few localities, but mostly with water depths between 37–30 meters (PG2209, PG2144.1, PG2117.1), show significant positive NRI values (Fig 5B). Generally, lineages of *Staurosira* seem to be more randomly distributed in Lake Bolshoe Toko indicating less signals of environmental filtering than *Aulacoseira*.

## Discussion

### Genetic and morphological pattern of diatom diversity within heterogenous lakes

The overall taxonomic composition obtained from genetic and morphological data reveals a distinct intra-lake diatom distribution pattern in the investigated localities of the large and heterogenous Lake Bolshoe Toko. Both approaches largely overlap in beta diversity estimations, whereas we find large differences in the taxonomic assignment and richness of identified taxa, similar to that reported in studies on stream invertebrates [57] or seagrass communities [58]. In our data, the families Fragilariacaea and Aulacoseiraceae are abundant and rich according to both approaches, but the genetic dataset does not uncover the abundance and diversity within the family Stephanodiscaceae and Achnanthaceae. However, overall total richness is greater in the metabarcoding approach compared to morphology. We detect an overrepresentation of diverse sequence types of the family Fragilariacaea which were attributed to ongoing diversification of the genera *Staurosira* and *Fragilaria* in lakes of the Siberian Arctic [26]. High genetic diversity in *Fragilaria* has also been found in a study from the 1990s indicating large differentiation in the species *F*. *crotonensis* along a latitudinal gradient from North to South American lakes formed since the Last Glacial [59]. Despite the great diversity in Fragilariacaea, the high genetic diversity within Aulacoseiraceae is unique in Bolshoe Toko and might be related to the overall high abundance and diversity of centric diatoms, mostly *Pliocaenicus bolshetokoensis*, *Cyclotella* sp. and *Aulacoseira* sp., as identified by morphological analysis. Although the marker can differentiate between the genera *Aulacoseira* and *Cyclotella*, sequence types that originate from dominant taxa like *Pliocaenicus bolshetokoensis* and *Cyclotella comensis* have no Genbank entries and occurrences in the genetic data are probably hidden behind sequence types which are only assigned to higher taxonomic levels than family (in total 68 sequence types, which represent about 20% of total reads) and thus are excluded from the final dataset. Nevertheless, part of the Aulacoseiraceae diversity is attributed to species (*Aulacoseira* sp.) and sub-species (*A*. *distance*, *A*. *subarctica*) variation (S2 Table), which is a first indication of genetic diversification within Lake Bolshoe Toko. *Aulacoseira subarctica*, for example, is able to form resting stages [60] which may promote the detectability of the genus in sedimentary DNA during summer. It has been reported that the formation of *Aulacoseira* resting spores starts by the end of the spring bloom, when phosphate concentrations decline, temperature rises and summer stratification establishes in the lakes [60,61]. During that time no spores occur in the water column but are buried in the sediments. With vertical mixing of the water column during autumn resting spores are resuspended and form new *Aulacoseira* populations during late autumn and winter [61].

We attribute the differences in alpha diversity between both approaches mainly to the incompleteness of the sequence reference database for diatom rbcL sequences [62,63], which is not ideal because sequence types will be lost when they do not cross the similarity threshold. In addition, variations in the taxonomic resolution of the rbcL marker in diatom genera or families might bias the depth of taxonomic assignment [24,26]. Despite strong filtering of raw sequences, we cannot exclude that a small part of the alpha diversity seen in our genetic dataset could be artificially created by technical biases from PCR and Illumina sequencing [64].

The beta diversity retrieved from both approaches is, in contrast to alpha diversity, largely similar. This aligns with previous results from other studies using rbcL, for example, coastal European sites [65] and Arctic lakes [24], and is also supported by surveys based on ribosomal metabarcodes applied to river [22] or marine diatom assemblages [66]. The similarity found in our study is most prominent in the local contribution to beta diversity (LCBD). Both approaches reveal the uniqueness of the same three localities, one close to the northern shore of Lake Bolshoe Toko and the others from the lagoon, a part which is gradually becoming separated from the main basin of the lake, putatively explaining their peculiarity [38]. We conclude that despite the differences in alpha diversity and the taxonomic assignment of both morphological and phylogenetic approaches, beta diversity displays a more similar intra-lake diatom community pattern in Lake Bolshoe Toko.

### Relationship between lake water depth and genetic and morphological intra-lake diatom diversity

Our study detects a strong relationship between intra-lake diatom diversity and water depth variation within the big glacial Lake Bolshoe Toko as suggested by [38]. Alpha and beta diversity estimates based on morphological investigations have a negative correlation with water depth. A decreasing richness related to increasing water depth has been found in other morphologically based diatom studies: for example, within-lake analysis of water-depth variations in Canadian Worth Lake [67], within Lugu Lake, China [68], in Lake Ohrid in Southern Europe [11] and across a spatial dataset of alpine lakes with varying water depth [69]. In general, variations in water depth are related to differences in substrate [70] and correlate with water turbulence [71]. Light and nutrient variability in lakes fosters shifts in diatom assembly due to their adaptation to different ecological conditions [67,72]. Shallower water conditions offer greater variability in habitat features formed e.g. by the presence of different substrates and macrophytes and thus life-form strategies which might explain the greater diversity found at low lake depths [67,73] compared to deep lake water conditions, which favor euplanktonic taxa adapted to limited light conditions. Our genetic and morphological data reflect general expectations in diatom community change along with water-depth variations. We detect a decrease of araphid and raphid pennate taxa with increasing water depth, whereas centric planktonic taxa rise. This shift is, however, more pronounced in the morphological data. We assume that unlimited light conditions between 0–10 m water depth support niches for motile epiphytic as well as passively moving planktonic or araphid diatoms. As light becomes limited by increasing water depth, benthic and epiphytic habitats vanish and the number of planktonic diatoms increases. Frequent turbulence transports fast-sinking centric diatoms, like *Aulacoseira*, to the photic zone. The discovery that habitat diversity plays a key role in the intra-specific variation in diatoms explicitly reviewed in [74], might partly explain the large intra-specific diversity in Fragilariaceae and Aulocaseiraceae in heterogeneous habitats within Lake Bolshoe Toko. Moreover, our genetic results based on the abundance of sequence types detect no influence of water depth on diatom alpha diversity. This might be attributed to the fact that the genetic approach is able to discern intra-specific or cryptic diversity, which might have been undetected via microscopy. Such disparity between morphology and genetics has also been detected in the centric freshwater diatom *Cyclotella meneghiana* on a small geographical scale [17].

The genetic beta diversity (LCBD), like the morphological beta diversity, revealed a negative relationship (Fig 3C) with water depth, implying less contribution from deep-water communities to diatom turnover. The similarity in beta diversity patterns is also demonstrated by the concordance of the ordination analyses of both approaches. A similar relationship between water depth and taxonomic turnover in morphological data has been reported in several case studies of surface samples in large lakes [67,75,76] as well as in a small boreal lake [16]. In our study, the proportion of explained variance based on water depth varies among the genetically and morphologically derived diatom compositions (24.2 and 15.81%, respectively). In both datasets the proportions of variance based on water depth compared to the proportions of PC1 likely explaining the variance of an unmeasured environmental variable. Perhaps this variance could be attributed to nutrient variability in, for example, total organic carbon (TOC) and total nitrogen (TN). Changes in TOC/TN ratio, as detected by [38], indicate the relative influx of land and water plants [77] and might lead to higher nutrient contents at river entries [70].

### Phylogenetic intra-lake diversity and its relation to lake water depth in diatom genera

In general, the phylogenetic diversity of sampled lake communities allows, in contrast to solely abundance-based diversity metrics, the incorporation of historical and evolutionary components of community structure [28] helping to explain current spatial patterns. We used the net relatedness index (NRI) to infer whether co-occurring sequence types within two specific diatom genera in within-lake locations differed in observed and expected mean phylogenetic diversity [30]. Our investigations on the *Staurosira* data set indicated rare signals of co-occurrence of closely related sequence types. There are only a few localities that show significant clustering. The genus *Aulacoseira*, however, shows that NRI values based on presence/absence data of sequence types are, in many cases, indicating a significant positive closer relatedness between co-occurring sequence types than in randomized communities, most strongly at localities of water depths between 30–37 meters. This signal is interpreted as environmental filtering, which arises from the adaptation of taxa to specific niche conditions. Environmental filtering has been reported for microbial communities [28,78,79], however patterns of clustering of closely-related sequence types might also result from differential dispersal or adaptive radiation events [78]. Adaptive radiation is simply defined as the divergence of closely related taxa into ecologically distinct lineages [80]. Adaptive radiation could be an explanation for the great biodiversity detected in Fragilariacaea and Aulacoseiraceae in the heterogeneous Lake Bolshoe Toko. This assumption is supported by the detection of strong environmental filtering of sequence types in the genus *Aulacoseira* in this study. However, there is only a tendency that water depth might force the clustering, we suggest that other environmental parameters, which were not tested in this study, might have a stronger influence. In the genus *Staurosira* we rarely detected environmental filtering implying that *Staurosira* sequence types are less ecologically adapted than *Aulacoseira* sequence types, which allow them to occur in high abundances in most of the sampled intra-lake localities. However, also *Staurosia* sequence types show environmental filtering for the localities with water depths between 30-37m as detected for *Aulacoseira*, which suggests that other environmental parameters, which were not tested in this study, might impact the abundance of specific *Aulacosiera* and *Staurosira* lineages.

Our study addresses the analyses of intra-lake diatom community variations along a lake water-depth gradient in the large sub-arctic Lake Bolshoe Toko. A combination of taxonomic (abundance-based) and phylogenetic diversity metrics applied to intra-lake diatom communities revealed (i) a distinct intra-lake diversity pattern, although genetic and morphological approaches show vast differences in taxonomic delineations and alpha diversity due to intra-specific or cryptic diversity, which might have been undetected via microscopy; (ii) that the current patterns are largely formed by lake water-depth variations or water-depth related environmental conditions, supporting a decrease in diversity with increasing water depth; and (iii) phylogenetic diversity patterns in two dominant diatom genera supported phylogenetic clustering in *Aulacosiera* mostly attributed to intermediate or greater lake depth, whereas *Staurosira* is less affected by environmental filtering. We speculate that the heterogeneity of the lake system affects diatom diversification as identified by distinct intra-lake diversity patterns obtained from a combination of taxonomic and phylogenetic diversity, suggesting Lake Bolshoe Toko represents a unique biodiversity hotspot in Yakutia.

## Supporting information

S1 Fig Results of the PROCRUSTES analyses. Procrustes error plots indicate A–the distance between diatom assembly data derived from genetic and morphological data, B–residuals of the comparison between PCA site scores derived of genetic and morphological data (residuals were ordered according to water depth of the lake, beginning from the shallowest site). Dashed and solid lines are the first, second and third quartiles.
(EPS)

S2 Fig Relationship between richness, water depth and NRI. Scatter plots show correlations between water depth and richness (a) and richness and NRI (from presence/absence (pa) data) (b) for the genus *Aulacoseira*. The plot (c) indicates the correlation between water depth and richness and (d) between richness and NRI (from presence/absence (pa) data) for the genus *Staurosira*.
(EPS)

S1 Table Sampling localities. Compilation of coordinates and water depth of the intra-lake sampling sites in Lake Bolshoe Toko.
(DOCX)

S2 Table Sequence types. Short names of identified sequence types (genetic dataset) and species (morphological dataset), sequence similarity, species contribution to beta diversity (SCBD) and relative abundance (%) in the total data set.
(DOCX)

S3 Table Valve counts. Total valve counts of morphologically identified diatoms in the 17 intra-lakes sites of Lake Bolshoe Toko.
(DOCX)

S4 Table Read counts. Total read counts of genetically identified diatoms in the 17 intra-lakes sites of Lake Bolshoe Toko.
(DOCX)

S5 Table Data compilation. Compilation of total sample count, rarefied sample count, water depth, taxonomic alpha diversity (richness and effective number of species) and phylogenetic diversity (NRI-Net relatedness index, pa-presence/absence data; aw-average weighted abundance data) for the genera *Aulacoseira* and *Staurosira* of the 17 intra-lake localities in Bolshoe Toko.
(DOCX)
