# Supplementary material for: Lake-depth related pattern of genetic and morphological diatom diversity in boreal Lake Bolshoe Toko, Eastern Siberia
Source: PLoS One. 2020 Apr 15;15(4):e0230284. doi: 10.1371/journal.pone.0230284 (PMC7159240; doi:10.1371/journal.pone.0230284)
Supplement: S1 Table — Compilation of coordinates and water depth of the intra-lake sampling sites in Lake Bolshoe Toko. (DOCX) [file pone.0230284.s003.docx]

**Table S1** Sampling localities, coordinates and water depth of the intra-lake sampling sites in Lake Bolshoe Toko.

| **Sampling**  **sites** | **Latitude (°)** | **Longitude (°)** | **water depth (m)** |
| --- | --- | --- | --- |
| PG2113-1 | 56.054 | 130.833 | 62.0 |
| PG2115-1 | 56.020 | 130.837 | 45.5 |
| PG2117-1 | 56.017 | 130.857 | 36.9 |
| PG2118-1 | 56.031 | 130.857 | 62.3 |
| PG2122-1 | 56.025 | 130.919 | 18.3 |
| PG2123-1 | 56.032 | 130.924 | 06.1 |
| PG2124 | 55.997 | 130.886 | 30.0 |
| PG2125 | 55.996 | 130.849 | 30.0 |
| PG2137-1 | 56.061 | 130.941 | 05.8 |
| PG2140-1 | 56.085 | 130.938 | 25.0 |
| PG2141-1 | 56.093 | 130.930 | 27.0 |
| PG2142-1 | 56.101 | 130.921 | 00.0 |
| PG2144-1 | 56.055 | 130.904 | 36.8 |
| PG2146-1 | 56.047 | 130.930 | 05.0 |
| PG2147-1 | 56.046 | 130.934 | 11.0 |
| PG2205-2 | 56.056 | 130.868 | 68.3 |
| PG2209-1 | 56.073 | 130.850 | 31.2 |
