# Supplementary material for: Lake-depth related pattern of genetic and morphological diatom diversity in boreal Lake Bolshoe Toko, Eastern Siberia
Source: PLoS One. 2020 Apr 15;15(4):e0230284. doi: 10.1371/journal.pone.0230284 (PMC7159240; doi:10.1371/journal.pone.0230284)
Supplement: S2 Table — Short names of identified sequence types (genetic dataset) and species (morphological dataset), sequence similarity, species contribution to beta diversity (SCBD) and relative abundance (%) in the total data set. (DOCX) [file pone.0230284.s004.docx]

**Table S2** Short names of identified sequence types (genetic dataset) and species (morphological dataset), sequence similarity, species contribution to beta diversity (SCBD) and relative abundance (%) in the total data set.

| **Short name** | **Sequence identity (%)** | **taxonomic rank** | **family name** | **genus name** | **species name** | **SCBD values** | **Relative abundance (%) from total data set** |
| --- | --- | --- | --- | --- | --- | --- | --- |
| **Genetic dataset** | | | | | | | |
| ach_1 | 100,0 | family | Achnanthidiaceae | NA | NA | 0,00511 | 0,026 |
| Amp_1 | 98,7 | genus | Catenulaceae | Amphora | NA | 0,00019 | 0,024 |
| Amp_2 | 98,7 | genus | Catenulaceae | Amphora | NA | 0,00031 | 0,018 |
| Amp_3 | 98,7 | genus | Catenulaceae | Amphora | NA | 0,00031 | 0,038 |
| Amp_4 | 97,4 | genus | Catenulaceae | Amphora | NA | 0,00026 | 0,045 |
| Amp_5 | 100,0 | genus | Catenulaceae | Amphora | NA | 0,01603 | 0,063 |
| Amp_6 | 94,7 | genus | Catenulaceae | Amphora | NA | 0,00017 | 0,025 |
| Amp_7 | 100,0 | genus | Catenulaceae | Amphora | NA | 0,02195 | 1,498 |
| Ast_1 | 93,5 | species | Fragilariaceae | Asterionella | Asterionella formosa | 0,00475 | 0,245 |
| Aul_1 | 97,4 | species | Aulacoseiraceae | Aulacoseira | Aulacoseira distans | 0,00006 | 0,014 |
| Aul_10 | 98,7 | species | Aulacoseiraceae | Aulacoseira | Aulacoseira distans | 0,00063 | 0,082 |
| Aul_11 | 96,1 | genus | Aulacoseiraceae | Aulacoseira | NA | 0,00303 | 0,463 |
| Aul_12 | 94,7 | genus | Aulacoseiraceae | Aulacoseira | NA | 0,00286 | 0,498 |
| Aul_13 | 96,1 | genus | Aulacoseiraceae | Aulacoseira | NA | 0,00006 | 0,014 |
| Aul_14 | 94,7 | genus | Aulacoseiraceae | Aulacoseira | NA | 0,00031 | 0,024 |
| Aul_15 | 96,1 | genus | Aulacoseiraceae | Aulacoseira | NA | 0,00029 | 0,035 |
| Aul_16 | 94,7 | genus | Aulacoseiraceae | Aulacoseira | NA | 0,00035 | 0,016 |
| Aul_17 | 94,7 | genus | Aulacoseiraceae | Aulacoseira | NA | 0,00046 | 0,047 |
| Aul_18 | 92,1 | genus | Aulacoseiraceae | Aulacoseira | NA | 0,00057 | 0,087 |
| Aul_19 | 97,4 | species | Aulacoseiraceae | Aulacoseira | Aulacoseira distans | 0,00139 | 0,200 |
| Aul_2 | 96,1 | genus | Aulacoseiraceae | Aulacoseira | NA | 0,00034 | 0,057 |
| Aul_20 | 96,1 | genus | Aulacoseiraceae | Aulacoseira | NA | 0,00014 | 0,015 |
| Aul_21 | 96,1 | genus | Aulacoseiraceae | Aulacoseira | NA | 0,00037 | 0,022 |
| Aul_22 | 97,4 | species | Aulacoseiraceae | Aulacoseira | Aulacoseira distans | 0,00027 | 0,024 |
| Aul_23 | 94,7 | genus | Aulacoseiraceae | Aulacoseira | NA | 0,00045 | 0,042 |
| Aul_24 | 96,1 | genus | Aulacoseiraceae | Aulacoseira | NA | 0,00047 | 0,057 |
| Aul_25 | 100,0 | genus | Aulacoseiraceae | Aulacoseira | NA | 0,00180 | 0,056 |
| Aul_26 | 93,4 | genus | Aulacoseiraceae | Aulacoseira | NA | 0,00226 | 0,129 |
| Aul_27 | 97,4 | species | Aulacoseiraceae | Aulacoseira | Aulacoseira distans | 0,02479 | 4,230 |
| Aul_28 | 94,7 | genus | Aulacoseiraceae | Aulacoseira | NA | 0,00024 | 0,025 |
| Aul_29 | 92,1 | genus | Aulacoseiraceae | Aulacoseira | NA | 0,00019 | 0,026 |
| Aul_3 | 94,7 | genus | Aulacoseiraceae | Aulacoseira | NA | 0,00083 | 0,137 |
| Aul_30 | 98,7 | genus | Aulacoseiraceae | Aulacoseira | NA | 0,00011 | 0,005 |
| Aul_31 | 96,1 | genus | Aulacoseiraceae | Aulacoseira | NA | 0,00032 | 0,017 |
| Aul_32 | 97,4 | species | Aulacoseiraceae | Aulacoseira | Aulacoseira subarctica | 0,00155 | 0,279 |
| Aul_33 | 96,1 | genus | Aulacoseiraceae | Aulacoseira | NA | 0,02926 | 5,767 |
| Aul_34 | 98,7 | genus | Aulacoseiraceae | Aulacoseira | NA | 0,00012 | 0,005 |
| Aul_35 | 98,7 | genus | Aulacoseiraceae | Aulacoseira | NA | 0,00050 | 0,017 |
| Aul_36 | 97,4 | species | Aulacoseiraceae | Aulacoseira | Aulacoseira distans | 0,00027 | 0,018 |
| Aul_37 | 94,7 | genus | Aulacoseiraceae | Aulacoseira | NA | 0,00036 | 0,019 |
| Aul_38 | 91,0 | genus | Aulacoseiraceae | Aulacoseira | NA | 0,00025 | 0,026 |
| Aul_39 | 98,7 | species | Aulacoseiraceae | Aulacoseira | Aulacoseira subarctica | 0,00070 | 0,023 |
| Aul_4 | 98,7 | genus | Aulacoseiraceae | Aulacoseira | NA | 0,00000 | 0,000 |
| Aul_40 | 98,7 | genus | Aulacoseiraceae | Aulacoseira | NA | 0,00044 | 0,014 |
| Aul_41 | 98,7 | genus | Aulacoseiraceae | Aulacoseira | NA | 0,00039 | 0,015 |
| Aul_42 | 97,4 | species | Aulacoseiraceae | Aulacoseira | Aulacoseira subarctica | 0,00061 | 0,032 |
| Aul_43 | 94,7 | genus | Aulacoseiraceae | Aulacoseira | NA | 0,00091 | 0,036 |
| Aul_44 | 97,4 | genus | Aulacoseiraceae | Aulacoseira | NA | 0,00074 | 0,041 |
| Aul_45 | 94,7 | genus | Aulacoseiraceae | Aulacoseira | NA | 0,00037 | 0,022 |
| Aul_46 | 97,4 | genus | Aulacoseiraceae | Aulacoseira | NA | 0,00091 | 0,046 |
| Aul_47 | 96,1 | genus | Aulacoseiraceae | Aulacoseira | NA | 0,00141 | 0,073 |
| Aul_48 | 97,4 | species | Aulacoseiraceae | Aulacoseira | Aulacoseira subarctica | 0,00241 | 0,440 |
| Aul_49 | 98,7 | genus | Aulacoseiraceae | Aulacoseira | NA | 0,00030 | 0,013 |
| Aul_5 | 94,7 | genus | Aulacoseiraceae | Aulacoseira | NA | 0,00019 | 0,036 |
| Aul_50 | 92,1 | genus | Aulacoseiraceae | Aulacoseira | NA | 0,00023 | 0,014 |
| Aul_51 | 96,1 | genus | Aulacoseiraceae | Aulacoseira | NA | 0,00056 | 0,064 |
| Aul_52 | 96,1 | genus | Aulacoseiraceae | Aulacoseira | NA | 0,00142 | 0,229 |
| Aul_53 | 100,0 | species | Aulacoseiraceae | Aulacoseira | Aulacoseira subarctica | 0,03203 | 0,752 |
| Aul_54 | 98,7 | genus | Aulacoseiraceae | Aulacoseira | NA | 0,00018 | 0,012 |
| Aul_55 | 94,7 | genus | Aulacoseiraceae | Aulacoseira | NA | 0,00021 | 0,022 |
| Aul_56 | 92,1 | genus | Aulacoseiraceae | Aulacoseira | NA | 0,00044 | 0,026 |
| Aul_57 | 93,4 | genus | Aulacoseiraceae | Aulacoseira | NA | 0,00031 | 0,025 |
| Aul_58 | 97,4 | genus | Aulacoseiraceae | Aulacoseira | NA | 0,00068 | 0,049 |
| Aul_59 | 100,0 | species | Aulacoseiraceae | Aulacoseira | Aulacoseira subarctica | 0,00260 | 0,079 |
| Aul_6 | 94,7 | genus | Aulacoseiraceae | Aulacoseira | NA | 0,00251 | 0,118 |
| Aul_60 | 97,4 | species | Aulacoseiraceae | Aulacoseira | Aulacoseira distans | 0,00209 | 0,190 |
| Aul_61 | 93,4 | genus | Aulacoseiraceae | Aulacoseira | NA | 0,00023 | 0,025 |
| Aul_62 | 94,7 | genus | Aulacoseiraceae | Aulacoseira | NA | 0,00030 | 0,039 |
| Aul_63 | 96,1 | genus | Aulacoseiraceae | Aulacoseira | NA | 0,00058 | 0,043 |
| Aul_64 | 96,1 | genus | Aulacoseiraceae | Aulacoseira | NA | 0,00055 | 0,077 |
| Aul_65 | 97,4 | species | Aulacoseiraceae | Aulacoseira | Aulacoseira subarctica | 0,00075 | 0,083 |
| Aul_66 | 93,4 | genus | Aulacoseiraceae | Aulacoseira | NA | 0,00091 | 0,089 |
| Aul_67 | 93,4 | genus | Aulacoseiraceae | Aulacoseira | NA | 0,00093 | 0,150 |
| Aul_68 | 96,1 | genus | Aulacoseiraceae | Aulacoseira | NA | 0,00153 | 0,185 |
| Aul_69 | 97,4 | species | Aulacoseiraceae | Aulacoseira | Aulacoseira subarctica | 0,00167 | 0,231 |
| Aul_7 | 96,1 | genus | Aulacoseiraceae | Aulacoseira | NA | 0,00015 | 0,019 |
| Aul_70 | 98,7 | species | Aulacoseiraceae | Aulacoseira | Aulacoseira distans | 0,02823 | 2,686 |
| Aul_71 | 100,0 | species | Aulacoseiraceae | Aulacoseira | Aulacoseira valida | 0,06810 | 4,996 |
| Aul_72 | 97,4 | species | Aulacoseiraceae | Aulacoseira | Aulacoseira subarctica | 0,00014 | 0,013 |
| Aul_73 | 97,4 | genus | Aulacoseiraceae | Aulacoseira | NA | 0,00034 | 0,023 |
| Aul_74 | 98,7 | genus | Aulacoseiraceae | Aulacoseira | NA | 0,00061 | 0,044 |
| Aul_75 | 96,1 | genus | Aulacoseiraceae | Aulacoseira | NA | 0,00112 | 0,100 |
| Aul_76 | 96,1 | genus | Aulacoseiraceae | Aulacoseira | NA | 0,01223 | 2,254 |
| Aul_77 | 97,4 | varietas | Aulacoseiraceae | Aulacoseira | Aulacoseira distans | 0,01448 | 2,440 |
| Aul_78 | 98,7 | species | Aulacoseiraceae | Aulacoseira | Aulacoseira subarctica | 0,02690 | 4,015 |
| Aul_8 | 93,4 | genus | Aulacoseiraceae | Aulacoseira | NA | 0,00032 | 0,056 |
| Aul_9 | 93,4 | genus | Aulacoseiraceae | Aulacoseira | NA | 0,00050 | 0,059 |
| Cha_1 | 94,7 | genus | Chaetocerotaceae | Chaetoceros | NA | 0,00046 | 0,065 |
| cmy_1 | 98,7 | family | Cymbellaceae | NA | NA | 0,00003 | 0,001 |
| cmy_2 | 97,4 | family | Cymbellaceae | NA | NA | 0,00258 | 0,014 |
| cmy_3 | 100,0 | family | Cymbellaceae | NA | NA | 0,00199 | 0,021 |
| cmy_4 | 98,7 | family | Cymbellaceae | NA | NA | 0,05761 | 0,232 |
| Enc_1 | 98,7 | genus | Cymbellaceae | Encyonema | NA | 0,00223 | 0,008 |
| Fra_1 | 100,0 | genus | Fragilariaceae | Fragilaria | NA | 0,00203 | 0,086 |
| fra_1 | 98,7 | family | Fragilariaceae | NA | NA | 0,00507 | 0,131 |
| Fra_10 | 98,7 | species | Fragilariaceae | Fragilaria | Fragilaria construens | 0,00027 | 0,327 |
| fra_10 | 98,6 | family | Fragilariaceae | Fragilaria | NA | 0,00266 | 0,021 |
| Fra_11 | 97,4 | species | Fragilariaceae | Fragilaria | Fragilaria construens | 0,00019 | 0,359 |
| fra_11 | 92,2 | family | Staurosiraceae | NA | NA | 0,00798 | 0,021 |
| Fra_12 | 97,4 | species | Fragilariaceae | Fragilaria | Fragilaria construens | 0,00041 | 0,059 |
| fra_12 | 93,4 | family | Fragilariaceae | NA | NA | 0,00075 | 0,025 |
| Fra_13 | 98,7 | genus | Fragilariaceae | Fragilaria | Fragilaria construens | 0,00180 | 0,081 |
| fra_13 | 93,4 | family | Fragilariaceae | NA | NA | 0,00383 | 0,108 |
| Fra_14 | 98,7 | species | Fragilariaceae | Fragilaria | Fragilaria construens | 0,00049 | 0,919 |
| fra_14 | 93,4 | family | Fragilariaceae | NA | NA | 0,00503 | 0,030 |
| Fra_15 | 97,4 | species | Fragilariaceae | Fragilaria | Fragilaria construens | 0,00089 | 2,780 |
| fra_15 | 92,1 | family | Fragilariaceae | NA | NA | 0,02083 | 0,039 |
| Fra_16 | 97,4 | species | Fragilariaceae | Fragilaria | Fragilaria construens | 0,00041 | 0,799 |
| fra_16 | 96,1 | family | Fragilariaceae | NA | NA | 0,00431 | 0,017 |
| fra_17 | 96,1 | family | Fragilariaceae | NA | NA | 0,00078 | 0,024 |
| fra_18 | 90,9 | family | Fragilariaceae | NA | NA | 0,00061 | 0,054 |
| fra_19 | 97,4 | family | Fragilariaceae | NA | NA | 0,00161 | 0,252 |
| Fra_2 | 97,4 | species | Fragilariaceae | Fragilaria | Fragilaria construens | 0,00045 | 0,014 |
| fra_2 | 96,1 | family | Fragilariaceae | NA | NA | 0,00150 | 0,195 |
| fra_20 | 93,5 | family | Fragilariaceae | NA | NA | 0,00021 | 0,015 |
| fra_21 | 94,7 | family | Fragilariaceae | NA | NA | 0,00035 | 0,046 |
| fra_22 | 94,7 | family | Fragilariaceae | NA | NA | 0,00661 | 0,227 |
| fra_23 | 97,4 | genus | Fragilariaceae | NA | NA | 0,00030 | 0,013 |
| fra_24 | 97,4 | genus | Fragilariaceae | NA | NA | 0,00288 | 0,133 |
| fra_25 | 96,1 | family | Fragilariaceae | NA | NA | 0,00028 | 0,013 |
| fra_26 | 96,1 | family | Fragilariaceae | NA | NA | 0,00050 | 0,025 |
| fra_27 | 96,1 | family | Fragilariaceae | NA | NA | 0,00026 | 0,069 |
| Fra_3 | 97,4 | species | Fragilariaceae | Fragilaria | Fragilaria construens | 0,00013 | 0,137 |
| fra_3 | 92,2 | family | Fragilariaceae | NA | NA | 0,00426 | 0,025 |
| Fra_4 | 97,4 | species | Fragilariaceae | Fragilaria | Fragilaria construens | 0,00011 | 0,018 |
| fra_4 | 89,6 | family | Fragilariaceae | NA | NA | 0,00063 | 0,014 |
| Fra_5 | 97,4 | species | Fragilariaceae | Fragilaria | Fragilaria construens | 0,00028 | 0,025 |
| fra_5 | 100,0 | family | Fragilariaceae | NA | NA | 0,00201 | 0,055 |
| Fra_6 | 98,7 | species | Fragilariaceae | Fragilaria | Fragilaria construens | 0,00014 | 0,090 |
| fra_6 | 94,7 | family | Fragilariaceae | NA | NA | 0,00274 | 0,016 |
| Fra_7 | 98,7 | species | Fragilariaceae | Fragilaria | Fragilaria construens | 0,00051 | 0,063 |
| fra_7 | 96,1 | family | Fragilariaceae | NA | NA | 0,00142 | 0,024 |
| Fra_8 | 100,0 | species | Fragilariaceae | Fragilaria | Fragilaria construens | 0,00301 | 0,632 |
| fra_8 | 96,1 | family | Fragilariaceae | NA | NA | 0,01609 | 0,097 |
| Fra_9 | 97,4 | species | Fragilariaceae | Fragilaria | Fragilaria construens | 0,00164 | 0,072 |
| fra_9 | 94,7 | family | Fragilariaceae | NA | NA | 0,00167 | 0,048 |
| Gom_1 | 98,7 | genus | Achnanthidiaceae | Achnanthidium | NA | 0,00302 | 0,089 |
| gom_1 | 98,7 | family | Gomphonemataceae | NA | NA | 0,01956 | 0,077 |
| Lem_1 | 97,4 | species | Achnanthidiaceae | Lemnicola | Lemnicola hungarica | 0,00301 | 0,083 |
| Mel_1 | 89,5 | genus | Melosiraceae | Melosira | NA | 0,00052 | 0,016 |
| nav_1 | 96,1 | family | Naviculaceae | NA | NA | 0,00883 | 0,084 |
| Nit_1 | 94,8 | genus | Bacillariaceae | Nitzschia | NA | 0,00078 | 0,123 |
| Pin_1 | 100,0 | genus | Pinnulariaceae | Pinnularia | NA | 0,00745 | 1,274 |
| Pin_2 | 100,0 | genus | Pinnulariaceae | Pinnularia | NA | 0,00043 | 0,049 |
| Pla_1 | 100,0 | genus | Achnanthaceae | Planothidium | NA | 0,00046 | 0,008 |
| Sel_1 | 100,0 | genus | Sellaphoraceae | Sellaphora | NA | 0,00041 | 0,018 |
| Sel_10 | 98,7 | genus | Sellaphoraceae | Sellaphora | Sellaphora cf. seminulum | 0,00260 | 0,151 |
| Sel_2 | 96,1 | genus | Sellaphoraceae | Sellaphora | NA | 0,00010 | 0,016 |
| Sel_3 | 97,4 | genus | Sellaphoraceae | Sellaphora | NA | 0,00120 | 0,035 |
| Sel_4 | 98,7 | genus | Sellaphoraceae | Sellaphora | NA | 0,00054 | 0,016 |
| Sel_5 | 100,0 | genus | Sellaphoraceae | Sellaphora | Sellaphora cf. seminulum | 0,00057 | 0,021 |
| Sel_6 | 94,7 | genus | Sellaphoraceae | Sellaphora | NA | 0,00154 | 0,081 |
| Sel_7 | 100,0 | species | Sellaphoraceae | Sellaphora | Sellaphora pupula | 0,00123 | 0,049 |
| Sel_8 | 96,1 | genus | Sellaphoraceae | Sellaphora | NA | 0,00291 | 0,043 |
| Sel_9 | 96,1 | genus | Sellaphoraceae | Sellaphora | NA | 0,00508 | 0,133 |
| Sta_1 | 97,4 | species | Staurosiraceae | Staurosira | Staurosira elliptica | 0,00091 | 0,023 |
| Sta_10 | 97,4 | species | Staurosiraceae | Staurosira | Staurosira elliptica | 0,00041 | 0,038 |
| Sta_11 | 97,4 | species | Staurosiraceae | Staurosira | Staurosira elliptica | 0,00143 | 0,068 |
| Sta_12 | 96,1 | species | Staurosiraceae | Staurosira | Staurosira elliptica | 0,00120 | 0,051 |
| Sta_13 | 96,1 | species | Staurosiraceae | Staurosira | Staurosira elliptica | 0,01241 | 0,348 |
| Sta_14 | 96,1 | species | Staurosiraceae | Staurosira | Staurosira elliptica | 0,00038 | 0,017 |
| Sta_15 | 98,7 | species | Staurosiraceae | Staurosira | Staurosira elliptica | 0,00118 | 0,046 |
| Sta_16 | 98,7 | species | Staurosiraceae | Staurosira | Staurosira elliptica | 0,00129 | 0,057 |
| Sta_17 | 100,0 | species | Staurosiraceae | Staurosira | Staurosira elliptica | 0,00386 | 0,126 |
| Sta_18 | 97,4 | species | Staurosiraceae | Staurosira | Staurosira elliptica | 0,00128 | 0,140 |
| Sta_19 | 98,7 | species | Staurosiraceae | Staurosira | Staurosira elliptica | 0,00531 | 0,365 |
| Sta_2 | 97,4 | species | Staurosiraceae | Staurosira | Staurosira elliptica | 0,00192 | 0,049 |
| Sta_20 | 96,1 | species | Staurosiraceae | Staurosira | Staurosira elliptica | 0,01301 | 0,626 |
| Sta_21 | 96,1 | species | Staurosiraceae | Staurosira | Staurosira elliptica | 0,00047 | 0,020 |
| Sta_22 | 97,4 | species | Staurosiraceae | Staurosira | Staurosira elliptica | 0,01894 | 1,954 |
| Sta_23 | 98,7 | species | Staurosiraceae | Staurosira | Staurosira elliptica | 0,01025 | 0,403 |
| Sta_24 | 97,4 | species | Staurosiraceae | Staurosira | Staurosira elliptica | 0,01509 | 0,851 |
| Sta_25 | 97,4 | species | Staurosiraceae | Staurosira | Staurosira elliptica | 0,04887 | 6,940 |
| Sta_3 | 97,4 | species | Staurosiraceae | Staurosira | Staurosira elliptica | 0,00058 | 0,015 |
| Sta_4 | 93,5 | species | Staurosiraceae | Staurosira | Staurosira elliptica | 0,00084 | 0,024 |
| Sta_5 | 97,4 | species | Staurosiraceae | Staurosira | Staurosira elliptica | 0,00049 | 0,014 |
| Sta_6 | 98,7 | species | Staurosiraceae | Staurosira | Staurosira elliptica | 0,00151 | 0,042 |
| Sta_7 | 96,1 | species | Staurosiraceae | Staurosira | Staurosira elliptica | 0,00027 | 0,010 |
| Sta_8 | 96,1 | species | Staurosiraceae | Staurosira | Staurosira elliptica | 0,00017 | 0,023 |
| Sta_9 | 98,7 | species | Staurosiraceae | Staurosira | Staurosira elliptica | 0,00029 | 0,013 |
| Stu_1 | 92,1 | species | Stauroneidaceae | Stauroneis | Stauroneis constricta | 0,00003 | 0,002 |
| Stu_10 | 92,1 | species | Stauroneidaceae | Stauroneis | Stauroneis constricta | 0,00017 | 0,012 |
| Stu_11 | 92,1 | species | Stauroneidaceae | Stauroneis | Stauroneis constricta | 0,00021 | 0,015 |
| Stu_12 | 92,1 | species | Stauroneidaceae | Stauroneis | Stauroneis constricta | 0,00021 | 0,019 |
| Stu_13 | 92,1 | species | Stauroneidaceae | Stauroneis | Stauroneis constricta | 0,00026 | 0,019 |
| Stu_14 | 92,1 | species | Stauroneidaceae | Stauroneis | Stauroneis constricta | 0,00022 | 0,022 |
| Stu_15 | 92,1 | species | Stauroneidaceae | Stauroneis | Stauroneis constricta | 0,00071 | 0,060 |
| Stu_16 | 93,4 | species | Stauroneidaceae | Stauroneis | Stauroneis constricta | 0,00018 | 0,018 |
| Stu_17 | 92,1 | species | Stauroneidaceae | Stauroneis | Stauroneis constricta | 0,00038 | 0,028 |
| Stu_18 | 92,1 | species | Stauroneidaceae | Stauroneis | Stauroneis constricta | 0,00046 | 0,038 |
| Stu_19 | 92,1 | species | Stauroneidaceae | Stauroneis | Stauroneis constricta | 0,00033 | 0,039 |
| Stu_2 | 96,1 | species | Stauroneidaceae | Stauroneis | Stauroneis constricta | 0,00026 | 0,016 |
| Stu_20 | 92,1 | species | Stauroneidaceae | Stauroneis | Stauroneis constricta | 0,00063 | 0,049 |
| Stu_21 | 92,1 | species | Stauroneidaceae | Stauroneis | Stauroneis constricta | 0,00051 | 0,048 |
| Stu_22 | 92,1 | species | Stauroneidaceae | Stauroneis | Stauroneis constricta | 0,00056 | 0,049 |
| Stu_23 | 92,1 | species | Stauroneidaceae | Stauroneis | Stauroneis constricta | 0,00057 | 0,057 |
| Stu_24 | 92,1 | species | Stauroneidaceae | Stauroneis | Stauroneis constricta | 0,00056 | 0,072 |
| Stu_25 | 92,1 | species | Stauroneidaceae | Stauroneis | Stauroneis constricta | 0,00043 | 0,067 |
| Stu_26 | 93,4 | species | Stauroneidaceae | Stauroneis | Stauroneis constricta | 0,00107 | 0,140 |
| Stu_27 | 94,7 | species | Stauroneidaceae | Stauroneis | Stauroneis constricta | 0,01228 | 2,047 |
| Stu_28 | 93,4 | species | Stauroneidaceae | Stauroneis | Stauroneis constricta | 0,26149 | 39,193 |
| Stu_29 | 92,1 | species | Stauroneidaceae | Stauroneis | Stauroneis constricta | 0,00058 | 0,066 |
| Stu_3 | 94,7 | species | Stauroneidaceae | Stauroneis | Stauroneis constricta | 0,00025 | 0,026 |
| Stu_30 | 94,7 | species | Stauroneidaceae | Stauroneis | Stauroneis constricta | 0,00060 | 0,076 |
| Stu_31 | 92,1 | species | Stauroneidaceae | Stauroneis | Stauroneis constricta | 0,00092 | 0,098 |
| Stu_4 | 93,5 | species | Stauroneidaceae | Stauroneis | Stauroneis constricta | 0,00023 | 0,020 |
| Stu_5 | 92,1 | species | Stauroneidaceae | Stauroneis | Stauroneis constricta | 0,00019 | 0,009 |
| Stu_6 | 92,1 | species | Stauroneidaceae | Stauroneis | Stauroneis constricta | 0,00028 | 0,020 |
| Stu_7 | 93,4 | species | Stauroneidaceae | Stauroneis | Stauroneis constricta | 0,00018 | 0,014 |
| Stu_8 | 92,1 | species | Stauroneidaceae | Stauroneis | Stauroneis constricta | 0,00030 | 0,018 |
| Stu_9 | 90,8 | species | Stauroneidaceae | Stauroneis | Stauroneis constricta | 0,00066 | 0,055 |
| Tab_1 | 96,1 | species | Tabellariaceae | Tabellaria | Tabellaria flocculosa | 0,00162 | 0,019 |
| **Morphological dataset** | | | | | | | |
| Ach_did | / | species | Achnanthaceae | Achnanthes | Achnanthes cf. didyma | 0,01136 | 0,365 |
| Ach_sch | / | species | Achnanthaceae | Achnanthes | Achnanthes chlidanos | 0,00110 | 0,035 |
| Ach_lev | / | species | Achnanthaceae | Achnanthes | Achnanthes levanderi | 0,00055 | 0,017 |
| Ach_pus | / | species | Achnanthaceae | Achnanthes | Achnanthes pusilla | 0,01039 | 0,632 |
| Ach_sp | / | genus | Achnanthaceae | Achnanthes | Achnanthes sp. | 0,01252 | 0,448 |
| Acn_min | / | species | Achnanthaceae | Achnanthidium | **Achnanthidium minutissimum** | 0,05696 | 12,719 |
| Amp_sp | / | species | Catenulaceae | Amphora | Amphora Ehr.Sp. | 0,00368 | 0,132 |
| Amp_ina | / | species | Catenulaceae | Amphora | Amphora inariensis | 0,00504 | 0,225 |
| Amp_ped | / | species | Catenulaceae | Amphora | Amphora pediculus | 0,00811 | 0,322 |
| Ane_tus | / | species | Mastogloiaceae | Aneumastus | Aneumastus tusculus | 0,00279 | 0,099 |
| Aul_amb | / | species | Aulacoseiraceae | Aulacoseira | Aulacoseira ambigua | 0,00721 | 0,280 |
| Aul_dis | / | species | Aulacoseiraceae | Aulacoseira | Aulacoseira distans | 0,01176 | 0,675 |
| Aul_sp | / | genus | Aulacoseiraceae | Aulacoseira | Aulacoseira sp. | 0,00099 | 0,034 |
| Aul_sub | / | species | Aulacoseiraceae | Aulacoseira | Aulacoseira subarctica | 0,01945 | 3,199 |
| Aul_val | / | species | Aulacoseiraceae | Aulacoseira | Aulacoseira valida | 0,00644 | 0,225 |
| Bra_vit | / | species | Brachysiraceae | Brachysira | Brachysira vitrea | 0,00765 | 0,628 |
| Cal_bac | / | species | Naviculacaea | Caloneis | Caloneis baccilum | 0,00236 | 0,084 |
| Cal_sil | / | species | Naviculacaea | Caloneis | Caloneis silicula | 0,00200 | 0,066 |
| Cav_cfcoc | / | species | Cavinulaceae | Cavinula | Cavinula cf. cocconeiformis | 0,00954 | 0,307 |
| Cav_coc | / | species | Cavinulaceae | Cavinula | Cavinula cocconeiformis | 0,00516 | 0,213 |
| Cav_jae | / | species | Cavinulaceae | Cavinula | Cavinula jaernefeltii | 0,00728 | 0,294 |
| Cav_pse | / | species | Cavinulaceae | Cavinula | Cavinula pseudoscutiformis | 0,00305 | 0,108 |
| Coc_pla | / | species | Cocconeidaceae | Cocconeis | Cocconeis placentula | 0,01512 | 0,665 |
| Coc_sp | / | genus | Cocconeidaceae | Cocconeis | Cocconeis sp. | 0,00101 | 0,032 |
| Cra_sp | / | genus | Stauroneidaceae | Craticula | Craticula sp. | 0,00285 | 0,102 |
| Cyc_com | / | species | Stephanodiscaceae | Cyclotella | Cyclotella comensis tripartita complex | 0,08652 | 11,850 |
| Cyc_cyc | / | species | Stephanodiscaceae | Cyclotella | Cyclotella cyclopuncta ocellata complex | 0,08033 | 11,134 |
| Cyl_sp | / | species | Stephanodiscaceae | Cyclotella | Cyclotella iris | 0,02170 | 3,137 |
| Cym_pro | / | species | Cymbellaceae | Cymbella | Cymbella proxima | 0,00523 | 0,258 |
| Cym_sp | / | genus | Cymbellaceae | Cymbella | Cymbella sp. | 0,00446 | 0,200 |
| Dia_mes | / | species | Fragilariaceae | Diatoma | Diatoma mesodon | 0,01260 | 0,558 |
| Dip_ell | / | species | Diploneidaceae | Diploneis | Diploneis elliptica | 0,00534 | 0,289 |
| Dip_pet | / | species | Diploneidaceae | Diploneis | Diploneis petersenii | 0,00055 | 0,017 |
| Enc_sil | / | species | Cymbellaceae | Encynoma | Encynoma silesiacum | 0,01030 | 1,147 |
| Enc_lun | / | species | Cymbellaceae | Encynoma | Encyonema lunatum | 0,00203 | 0,068 |
| Enc_min | / | species | Cymbellaceae | Encynoma | Encyonema minutum | 0,00759 | 0,451 |
| Enc_sp | / | species | Cymbellaceae | Encynoma | Encyonema sp. | 0,00315 | 0,105 |
| Euc_fle | / | species | Achnanthidiaceae | Eucocconeis | Eucocconeis flexella | 0,00900 | 0,848 |
| Euc_lae | / | species | Achnanthidiaceae | Eucocconeis | Eucocconeis laevis | 0,01759 | 0,857 |
| Eun_bil | / | species | Eunotiaceae | Eunotia | Eunotia bilunaris | 0,00641 | 0,472 |
| Eun_den | / | species | Eunotiaceae | Eunotia | Eunotia denticulata | 0,00083 | 0,026 |
| Eun_min | / | species | Eunotiaceae | Eunotia | Eunotia minor | 0,00169 | 0,053 |
| Eun_pra | / | species | Eunotiaceae | Eunotia | Eunotia praerupta | 0,00506 | 0,228 |
| Eun_ser | / | species | Eunotiaceae | Eunotia | Eunotia serra | 0,00344 | 0,123 |
| Eun_sp | / | genus | Eunotiaceae | Eunotia | Eunotia sp. | 0,01404 | 0,700 |
| Fra_cap | / | species | Fragilariaceae | Fragilaria | Fragilaria capucina | 0,03460 | 3,396 |
| Fra_cfdel | / | species | Fragilariaceae | Fragilaria | Fragilaria cf. delicatissima | 0,00110 | 0,035 |
| Fra_cfper | / | species | Fragilariaceae | Fragilaria | Fragilaria cf. perminuta | 0,00215 | 0,067 |
| Fra_gra | / | species | Fragilariaceae | Fragilaria | Fragilaria graciles | 0,00112 | 0,035 |
| Fra_par | / | species | Fragilariaceae | Fragilaria | Fragilaria parasiticaet var. subconstricta | 0,00107 | 0,034 |
| Fra_pin | / | species | Fragilariaceae | Fragilaria | Fragilaria pinnata | 0,04001 | 2,257 |
| Fra_sp | / | genus | Fragilariaceae | Fragilaria | Fragilaria sp. | 0,00656 | 0,429 |
| Fra_vir | / | species | Fragilariaceae | Fragilaria | Fragilaria virescens | 0,00056 | 0,018 |
| Fru_rho | / | species | Amphipleuraceae | Frustulia | Frustulia rhomboides | 0,00304 | 0,116 |
| Gom_acu | / | species | Gomphonemataceae | Gomphonema | Gomphonema acuminatum | 0,00478 | 0,168 |
| Gom_ang | / | species | Gomphonemataceae | Gomphonema | Gomphonema angustatum | 0,00697 | 0,368 |
| Gom_gra | / | species | Gomphonemataceae | Gomphonema | Gomphonema gracile | 0,00722 | 0,350 |
| Gom_ins | / | species | Gomphonemataceae | Gomphonema | Gomphonema insigne | 0,00848 | 0,407 |
| Gom_sp | / | species | Gomphonemataceae | Gomphonema | Gomphonema sp. | 0,01353 | 0,685 |
| Han_arc | / | species | Fragilariaceae | Hannaea | Hannaea arcus | 0,00383 | 0,128 |
| Hip_cos | / | species | Naviculacaea | Hippodonta | Hippodonta costulata | 0,00629 | 0,234 |
| Kar_cfamo | / | species | Achnanthidiaceae | Karayevia | Karayevia cf. amoena | 0,00258 | 0,085 |
| Kar_lat | / | species | Achnanthidiaceae | Karayevia | Karayevia laterostrata | 0,02955 | 1,907 |
| Kar_suc | / | species | Achnanthidiaceae | Karayevia | Karayevia suchlandtii | 0,01522 | 0,671 |
| May_ato | / | species | Naviculacaea | Mayamaea | Mayamaea atomus | 0,00432 | 0,135 |
| Mer_cir | / | species | Fragilariaceae | Meridion | Meridion circulare | 0,00100 | 0,031 |
| Nav_cry | / | species | Naviculacaea | Navicula | Navicula cryptocephala | 0,00429 | 0,153 |
| Nav_rad | / | species | Naviculacaea | Navicula | Navicula radiosa | 0,00949 | 0,628 |
| Nav_rhy | / | species | Naviculacaea | Navicula | Navicula rhynchocephala | 0,00322 | 0,124 |
| Nav_sp | / | genus | Naviculacaea | Navicula | Navicula sp. | 0,00989 | 0,695 |
| Nei_aff | / | species | Neidiaceae | Neidium | Neidium affine | 0,00108 | 0,034 |
| Nei_bis | / | species | Neidiaceae | Neidium | Neidium bisulcatum | 0,00102 | 0,032 |
| Nei_lad | / | species | Neidiaceae | Neidium | Neidium ladogensis | 0,00101 | 0,032 |
| Nei_sp | / | genus | Neidiaceae | Neidium | Neidum sp. | 0,00056 | 0,018 |
| Niz_ang | / | species | Bacillariaceae | Nitzschia | Nitzschia angustata | 0,00782 | 0,322 |
| Niz_dis | / | species | Bacillariaceae | Nitzschia | Nitzschia dissipata | 0,00761 | 1,735 |
| Niz_sp | / | genus | Bacillariaceae | Nitzschia | Nitzschia sp. | 0,01074 | 1,111 |
| Per_fib | / | species | Peroniaceae | Peronia | Peronia fibula | 0,00463 | 0,191 |
| Pin_bor | / | species | Pinnulariaceae | Pinnularia | Pinnularia borealis | 0,00158 | 0,052 |
| Pin_mar | / | species | Pinnulariaceae | Pinnularia | Pinnularia marchica | 0,00455 | 0,148 |
| Pin_nod | / | species | Pinnulariaceae | Pinnularia | Pinnularia nodosa | 0,00209 | 0,065 |
| Pla_cfpla | / | species | Cymbellaceae | Placoneis | Placoneis cf. placentula | 0,00112 | 0,035 |
| Pla_min | / | species | Cymbellaceae | Placoneis | Placoneis minor | 0,00108 | 0,034 |
| Pln_cal | / | species | Achnanthidiaceae | Planothidium | Planothidium calcar | 0,00694 | 0,307 |
| Pln_lan | / | species | Achnanthidiaceae | Planothidium | Planothidium lanceolata | 0,00665 | 0,285 |
| Pln_est | / | species | Achnanthidiaceae | Planothidium | Planothidium oestrupii | 0,00918 | 0,338 |
| Pln_per | / | species | Achnanthidiaceae | Planothidium | Planothidium peragalli | 0,00215 | 0,067 |
| Pla_sp | / | species | Achnanthidiaceae | Planothidium | Planothidium sp. | 0,00104 | 0,035 |
| Pli_bol | / | species | Stephanodiscaceae | Pliocaenicus | Pliocaenicus bolshetokoensis | 0,03906 | 16,160 |
| Psm_bio | / | species | Achnanthaceae | Psammothidium | Psammothidium bioretti | 0,03051 | 2,659 |
| Psm_hel | / | species | Achnanthaceae | Psammothidium | Psammothidium helveticum | 0,00262 | 0,088 |
| Psm_lev | / | species | Achnanthaceae | Psammothidium | Psammothidium levanderi | 0,00400 | 0,138 |
| Psm_mar | / | species | Achnanthaceae | Psammothidium | Psammothidium marginulatum | 0,00212 | 0,070 |
| Psm_ros | / | species | Achnanthaceae | Psammothidium | Psammothidium rossii | 0,00055 | 0,017 |
| Psm_sub | / | species | Achnanthaceae | Psammothidium | Psammothidium subatomoides | 0,00605 | 0,214 |
| Pse_abr | / | species | Fragilariaceae | Pseudostaurosira | Pseudostaurosira brevistriata | 0,00770 | 0,270 |
| Rei_inu | / | species | Gomphonemataceae | Reimeria | Reimeria sinuata | 0,02183 | 0,928 |
| Rho_sp | / | species | Rhopalodiaceae | Rhopalodia | Rhopalodia sp. | 0,00107 | 0,034 |
| Sel_bac | / | species | Sellaphoraceae | Sellaphora | Sellaphora bacillum | 0,00107 | 0,034 |
| Sel_cfpse | / | species | Sellaphoraceae | Sellaphora | Sellaphora cf. pseudopupula | 0,00108 | 0,034 |
| Sel_pup | / | species | Sellaphoraceae | Sellaphora | Sellaphora pupula | 0,00433 | 0,165 |
| Stu_anc | / | species | Stauroneidaceae | Stauroneis | Stauroneis anceps | 0,00244 | 0,087 |
| Stu_pho | / | species | Stauroneidaceae | Stauroneis | Stauroneis phoenicenteron | 0,00105 | 0,033 |
| Sta_con | / | species | Fragilariaceae | Staurosira | Staurosira construens | 0,00479 | 0,169 |
| Sta_ven | / | species | Fragilariaceae | Staurosira | Staurosira venter | 0,02566 | 0,804 |
| Sur_sp | / | species | Surirellaceae | Surirella | Surirella turp. sp. | 0,00105 | 0,033 |
| Tab_fen | / | species | Tabellareaceae | Tabellaria | Tabellaria fenestrata | 0,01365 | 1,661 |
| Tab_flo | / | species | Tabellareaceae | Tabellaria | Tabellaria flocculosa | 0,02271 | 3,890 |
| Tet_gla | / | species | Tabellareaceae | Tetracyclus | Tetracyclus glans | 0,00269 | 0,089 |
