# Supplementary material for: Lake-depth related pattern of genetic and morphological diatom diversity in boreal Lake Bolshoe Toko, Eastern Siberia
Source: PLoS One. 2020 Apr 15;15(4):e0230284. doi: 10.1371/journal.pone.0230284 (PMC7159240; doi:10.1371/journal.pone.0230284)
Supplement: S3 Table — Total valve counts of morphologically identified diatoms in the 17 intra-lakes sites of Lake Bolshoe Toko. (DOCX) [file pone.0230284.s005.docx]

**Table S3** Total valve counts of morphologically identified diatoms in the 17 intra-lakes sites of Lake Bolshoe Toko.

|  | **Lake sites** | | | | | | | | | | | | | | | | |
| --- | --- | --- | --- | --- | --- | --- | --- | --- | --- | --- | --- | --- | --- | --- | --- | --- | --- |
| **Taxon** | **PG 2113-1** | **PG 2115-1** | **PG 2117-1** | **PG 2118-1** | **PG 2122-1** | **PG 2123-1** | **PG 2124** | **PG 2125** | **PG 2137-1** | **PG 2140-1** | **PG 2141-1** | **PG 2142-1** | **PG 2144-1** | **PG 2146-1** | **PG 2147-1** | **PG 2205-2** | **PG 2209-1** |
| Achnanthes cf. didyma | 0 | 0 | 0 | 0 | 0 | 0 | 0 | 0 | 0 | 0 | 0 | 18 | 0 | 1 | 0 | 0 | 0 |
| Achnanthes chlidanos | 0 | 0 | 0 | 0 | 0 | 0 | 0 | 0 | 0 | 0 | 0 | 0 | 0 | 2 | 0 | 0 | 0 |
| Achnanthes levanderi | 0 | 0 | 0 | 0 | 0 | 0 | 0 | 0 | 0 | 0 | 0 | 0 | 0 | 1 | 0 | 0 | 0 |
| Achnanthes pusilla | 2 | 0 | 0 | 0 | 6 | 10 | 0 | 2 | 2 | 2 | 1 | 0 | 2 | 7 | 0 | 0 | 4 |
| Achnanthes sp. | 0 | 0 | 0 | 0 | 2 | 19 | 0 | 0 | 0 | 0 | 0 | 4 | 0 | 1 | 0 | 0 | 0 |
| Achnanthidium minutissimum | 26 | 39 | 132 | 38 | 0 | 17 | 53 | 82 | 51 | 58 | 23 | 38 | 46 | 40 | 96 | 47 | 8 |
| Amphora Ehr. Sp. | 0 | 0 | 2 | 0 | 0 | 0 | 0 | 0 | 0 | 0 | 0 | 0 | 0 | 0 | 0 | 4 | 2 |
| Amphora inariensis | 0 | 2 | 4 | 0 | 0 | 2 | 0 | 0 | 2 | 0 | 0 | 0 | 0 | 0 | 2 | 0 | 2 |
| Amphora pediculus | 0 | 0 | 0 | 0 | 0 | 2 | 0 | 0 | 10 | 0 | 0 | 2 | 0 | 0 | 0 | 2 | 3 |
| Aneumastus tusculus | 0 | 0 | 0 | 0 | 2 | 0 | 2 | 0 | 0 | 0 | 0 | 0 | 2 | 0 | 0 | 0 | 0 |
| Aulacoseira ambigua | 0 | 0 | 0 | 0 | 0 | 1 | 0 | 0 | 0 | 1 | 2 | 0 | 0 | 0 | 0 | 3 | 10 |
| Aulacoseira distans | 0 | 0 | 0 | 4 | 0 | 3 | 6 | 0 | 2 | 6 | 0 | 0 | 1 | 0 | 4 | 10 | 6 |
| Aulacoseira sp. | 0 | 0 | 0 | 0 | 0 | 0 | 0 | 0 | 0 | 0 | 1 | 0 | 1 | 0 | 0 | 0 | 0 |
| Aulacoseira subarctica | 15 | 14 | 9 | 24 | 20 | 1 | 17 | 12 | 11 | 3 | 1 | 0 | 15 | 4 | 7 | 21 | 23 |
| Aulacoseira valida | 0 | 0 | 0 | 0 | 0 | 0 | 0 | 0 | 0 | 0 | 6 | 0 | 1 | 6 | 0 | 0 | 0 |
| Brachysira vitrea | 2 | 0 | 0 | 2 | 2 | 0 | 0 | 2 | 4 | 2 | 1 | 0 | 4 | 3 | 13 | 4 | 2 |
| Caloneis baccilum | 0 | 0 | 2 | 2 | 0 | 0 | 0 | 0 | 0 | 0 | 0 | 0 | 0 | 1 | 0 | 0 | 0 |
| Caloneis silicula | 0 | 0 | 0 | 0 | 0 | 0 | 0 | 0 | 0 | 0 | 0 | 0 | 0 | 1 | 0 | 0 | 3 |
| Cavinula cf. cocconeiformis | 0 | 0 | 0 | 0 | 0 | 0 | 0 | 0 | 0 | 0 | 0 | 15 | 0 | 1 | 0 | 0 | 0 |
| Cavinula cocconeiformis | 2 | 0 | 0 | 0 | 0 | 0 | 0 | 0 | 2 | 0 | 0 | 4 | 0 | 2 | 0 | 0 | 2 |
| Cavinula jaernefeltii | 0 | 0 | 0 | 0 | 0 | 6 | 0 | 0 | 4 | 0 | 0 | 4 | 2 | 1 | 0 | 0 | 0 |
| Cavinula pseudoscutiformis | 0 | 0 | 0 | 0 | 0 | 0 | 0 | 0 | 0 | 0 | 0 | 2 | 0 | 1 | 4 | 0 | 0 |
| Cocconeis placentula | 0 | 2 | 0 | 0 | 16 | 12 | 0 | 0 | 4 | 0 | 0 | 0 | 2 | 0 | 2 | 0 | 2 |
| Cocconeis sp. | 0 | 0 | 0 | 0 | 0 | 0 | 0 | 0 | 0 | 2 | 0 | 0 | 0 | 0 | 0 | 0 | 0 |
| Craticula sp. | 0 | 0 | 2 | 0 | 0 | 2 | 0 | 0 | 0 | 0 | 0 | 0 | 0 | 2 | 0 | 0 | 0 |
| Cyclotella - comensis - tripartita - Komplex | 46 | 50 | 29 | 54 | 0 | 0 | 32 | 82 | 9 | 31 | 68 | 0 | 87 | 70 | 66 | 57 | 63 |
| Cyclotella - cyclopuncta - ocellata - Komplex | 60 | 47 | 10 | 46 | 0 | 0 | 55 | 76 | 19 | 76 | 40 | 0 | 71 | 34 | 46 | 66 | 53 |
| Cyclotella iris | 16 | 19 | 2 | 10 | 2 | 0 | 5 | 14 | 5 | 24 | 14 | 0 | 22 | 9 | 26 | 23 | 8 |
| Cymbella proxima | 0 | 0 | 2 | 0 | 2 | 4 | 2 | 2 | 0 | 0 | 0 | 0 | 0 | 2 | 2 | 0 | 0 |
| Cymbella sp. | 0 | 0 | 0 | 0 | 2 | 0 | 0 | 0 | 2 | 0 | 2 | 0 | 2 | 1 | 0 | 0 | 3 |
| Diatoma mesodon | 4 | 4 | 10 | 4 | 0 | 0 | 8 | 4 | 0 | 0 | 0 | 0 | 0 | 0 | 0 | 0 | 0 |
| Diploneis elliptica | 0 | 2 | 0 | 2 | 0 | 2 | 0 | 2 | 2 | 4 | 1 | 0 | 0 | 0 | 0 | 0 | 3 |
| Diploneis petersenii | 0 | 0 | 0 | 0 | 0 | 0 | 0 | 0 | 0 | 0 | 0 | 0 | 0 | 1 | 0 | 0 | 0 |
| Encynoma silesiacum | 4 | 4 | 8 | 8 | 0 | 4 | 0 | 6 | 0 | 2 | 4 | 0 | 6 | 5 | 13 | 4 | 5 |
| Encyonema lunatum | 0 | 0 | 0 | 0 | 2 | 2 | 0 | 0 | 0 | 0 | 0 | 0 | 0 | 0 | 0 | 0 | 0 |
| Encyonema minutum | 4 | 0 | 0 | 4 | 2 | 2 | 0 | 2 | 5 | 0 | 0 | 4 | 0 | 2 | 2 | 0 | 0 |
| Encyonema sp. | 0 | 0 | 0 | 0 | 0 | 0 | 0 | 0 | 0 | 0 | 4 | 0 | 0 | 2 | 0 | 0 | 0 |
| Eucocconeis flexella | 0 | 4 | 2 | 4 | 0 | 5 | 4 | 2 | 13 | 2 | 2 | 2 | 0 | 6 | 2 | 0 | 3 |
| Eucocconeis laevis | 0 | 0 | 0 | 0 | 0 | 8 | 2 | 0 | 16 | 2 | 0 | 12 | 4 | 0 | 2 | 0 | 4 |
| Eunotia bilunaris | 4 | 4 | 6 | 2 | 2 | 0 | 0 | 2 | 3 | 0 | 1 | 0 | 0 | 1 | 2 | 0 | 2 |
| Eunotia denticulata | 0 | 0 | 0 | 0 | 0 | 0 | 0 | 0 | 0 | 0 | 0 | 0 | 0 | 0 | 2 | 0 | 0 |
| Eunotia minor | 0 | 0 | 0 | 0 | 0 | 0 | 0 | 0 | 0 | 0 | 3 | 0 | 0 | 0 | 0 | 0 | 0 |
| Eunotia praerupta | 0 | 2 | 0 | 0 | 2 | 0 | 0 | 2 | 4 | 2 | 0 | 0 | 0 | 2 | 0 | 0 | 0 |
| Eunotia serra | 2 | 0 | 2 | 0 | 0 | 0 | 0 | 4 | 0 | 0 | 0 | 0 | 0 | 0 | 0 | 0 | 0 |
| Eunotia sp. | 0 | 0 | 4 | 0 | 13 | 6 | 0 | 0 | 0 | 0 | 0 | 0 | 1 | 1 | 5 | 5 | 8 |
| Fragilaria capucina | 12 | 32 | 50 | 10 | 12 | 13 | 24 | 22 | 4 | 6 | 3 | 0 | 0 | 5 | 0 | 4 | 10 |
| Fragilaria cf. delicatissima | 0 | 0 | 0 | 0 | 0 | 0 | 0 | 0 | 0 | 0 | 0 | 0 | 0 | 2 | 0 | 0 | 0 |
| Fragilaria cf. perminuta | 0 | 0 | 0 | 0 | 0 | 4 | 0 | 0 | 0 | 0 | 0 | 0 | 0 | 0 | 0 | 0 | 0 |
| Fragilaria graciles | 0 | 0 | 0 | 0 | 0 | 0 | 0 | 0 | 0 | 0 | 2 | 0 | 0 | 0 | 0 | 0 | 0 |
| Fragilaria parasitica et var. subconstricta | 0 | 0 | 0 | 0 | 0 | 2 | 0 | 0 | 0 | 0 | 0 | 0 | 0 | 0 | 0 | 0 | 0 |
| Fragilaria pinnata | 4 | 0 | 8 | 0 | 28 | 59 | 0 | 4 | 10 | 0 | 5 | 0 | 0 | 4 | 8 | 2 | 4 |
| Fragilaria sp. | 4 | 2 | 1 | 0 | 4 | 0 | 2 | 0 | 3 | 0 | 2 | 0 | 0 | 1 | 2 | 0 | 5 |
| Fragilaria virescens | 0 | 0 | 0 | 0 | 0 | 0 | 0 | 0 | 0 | 0 | 1 | 0 | 0 | 0 | 0 | 0 | 0 |
| Frustulia rhomboides | 0 | 0 | 2 | 0 | 0 | 0 | 0 | 0 | 0 | 0 | 1 | 0 | 3 | 1 | 0 | 0 | 0 |
| Gomphonema acuminatum | 0 | 2 | 0 | 0 | 6 | 2 | 0 | 0 | 0 | 0 | 0 | 0 | 0 | 0 | 0 | 0 | 0 |
| Gomphonema angustatum | 2 | 4 | 2 | 0 | 2 | 0 | 0 | 8 | 2 | 0 | 0 | 0 | 0 | 0 | 2 | 2 | 0 |
| Gomphonema gracile | 2 | 4 | 0 | 0 | 2 | 2 | 0 | 0 | 6 | 0 | 0 | 0 | 2 | 3 | 0 | 0 | 0 |
| Gomphonema insigne | 2 | 0 | 2 | 0 | 6 | 4 | 6 | 0 | 2 | 0 | 0 | 0 | 0 | 0 | 0 | 0 | 2 |
| Gomphonema sp. | 0 | 4 | 0 | 0 | 0 | 4 | 2 | 0 | 0 | 2 | 9 | 0 | 2 | 12 | 7 | 0 | 0 |
| Hannaea arcus | 0 | 0 | 0 | 4 | 0 | 0 | 0 | 0 | 0 | 0 | 0 | 0 | 4 | 0 | 0 | 0 | 0 |
| Hippodonta costulata | 0 | 0 | 0 | 0 | 0 | 0 | 2 | 0 | 8 | 0 | 0 | 2 | 0 | 0 | 2 | 0 | 0 |
| Karayevia cf. amoena | 0 | 0 | 0 | 0 | 0 | 0 | 0 | 0 | 4 | 0 | 1 | 0 | 0 | 0 | 0 | 0 | 0 |
| Karayevia laterostrata | 0 | 8 | 2 | 0 | 14 | 6 | 1 | 0 | 12 | 4 | 5 | 45 | 3 | 1 | 5 | 2 | 0 |
| Karayevia suchlandtii | 0 | 0 | 0 | 4 | 7 | 4 | 0 | 3 | 0 | 2 | 0 | 17 | 0 | 1 | 0 | 0 | 0 |
| Mayamaea atomus | 0 | 0 | 0 | 0 | 0 | 0 | 0 | 0 | 8 | 0 | 0 | 0 | 0 | 0 | 0 | 0 | 0 |
| Meridion circulare | 0 | 0 | 0 | 0 | 0 | 0 | 0 | 0 | 0 | 0 | 0 | 0 | 2 | 0 | 0 | 0 | 0 |
| Navicula cryptocephala | 0 | 0 | 0 | 0 | 0 | 0 | 0 | 0 | 0 | 2 | 0 | 0 | 0 | 4 | 4 | 0 | 0 |
| Navicula radiosa | 2 | 2 | 0 | 2 | 0 | 0 | 2 | 6 | 7 | 4 | 0 | 0 | 0 | 3 | 0 | 4 | 7 |
| Navicula rhynchocephala | 0 | 0 | 0 | 2 | 0 | 0 | 2 | 0 | 0 | 0 | 1 | 2 | 0 | 0 | 0 | 0 | 0 |
| Navicula sp. | 10 | 0 | 6 | 0 | 0 | 2 | 0 | 0 | 2 | 4 | 1 | 2 | 4 | 1 | 7 | 0 | 4 |
| Neidium affine | 0 | 0 | 2 | 0 | 0 | 0 | 0 | 0 | 0 | 0 | 0 | 0 | 0 | 0 | 0 | 0 | 0 |
| Neidium bisulcatum | 0 | 2 | 0 | 0 | 0 | 0 | 0 | 0 | 0 | 0 | 0 | 0 | 0 | 0 | 0 | 0 | 0 |
| Neidium ladogensis | 0 | 0 | 0 | 0 | 0 | 0 | 0 | 0 | 0 | 2 | 0 | 0 | 0 | 0 | 0 | 0 | 0 |
| Neidum sp. | 0 | 0 | 0 | 0 | 0 | 0 | 0 | 0 | 0 | 0 | 1 | 0 | 0 | 0 | 0 | 0 | 0 |
| Nitzschia angustata | 2 | 0 | 0 | 0 | 0 | 0 | 0 | 0 | 4 | 0 | 0 | 0 | 3 | 4 | 8 | 0 | 0 |
| Nitzschia dissipata | 10 | 4 | 4 | 4 | 0 | 8 | 2 | 4 | 16 | 10 | 6 | 10 | 5 | 6 | 8 | 2 | 6 |
| Nitzschia sp. | 6 | 10 | 10 | 4 | 5 | 6 | 0 | 2 | 5 | 4 | 1 | 0 | 5 | 0 | 7 | 4 | 0 |
| Peronia fibula | 0 | 2 | 4 | 0 | 0 | 0 | 0 | 0 | 0 | 0 | 0 | 0 | 0 | 0 | 2 | 2 | 2 |
| Pinnularia borealis | 0 | 0 | 0 | 0 | 0 | 0 | 0 | 0 | 0 | 0 | 2 | 0 | 0 | 1 | 0 | 0 | 0 |
| Pinnularia marchica | 0 | 0 | 0 | 8 | 0 | 0 | 0 | 0 | 0 | 0 | 0 | 0 | 0 | 1 | 0 | 0 | 0 |
| Pinnularia nodosa | 0 | 0 | 0 | 0 | 0 | 0 | 0 | 0 | 0 | 0 | 0 | 0 | 0 | 0 | 0 | 0 | 4 |
| Placoneis cf. placentula | 0 | 0 | 0 | 0 | 0 | 0 | 0 | 0 | 0 | 0 | 2 | 0 | 0 | 0 | 0 | 0 | 0 |
| Placoneis minor | 0 | 0 | 0 | 0 | 0 | 0 | 0 | 0 | 2 | 0 | 0 | 0 | 0 | 0 | 0 | 0 | 0 |
| Planothidium calcar | 0 | 0 | 0 | 0 | 0 | 4 | 0 | 0 | 4 | 4 | 1 | 0 | 0 | 0 | 2 | 0 | 4 |
| Planothidium lanceolata | 0 | 0 | 0 | 0 | 0 | 2 | 4 | 0 | 0 | 2 | 1 | 6 | 0 | 1 | 0 | 0 | 0 |
| Planothidium oestrupii | 0 | 0 | 0 | 0 | 0 | 8 | 0 | 0 | 0 | 0 | 0 | 8 | 2 | 1 | 0 | 0 | 0 |
| Planothidium peragalli | 0 | 0 | 0 | 0 | 0 | 4 | 0 | 0 | 0 | 0 | 0 | 0 | 0 | 0 | 0 | 0 | 0 |
| Planothidium sp. | 0 | 0 | 0 | 0 | 0 | 0 | 0 | 0 | 0 | 0 | 1 | 0 | 0 | 1 | 0 | 0 | 0 |
| Pliocaenicus bolshetokoensis | 86 | 78 | 12 | 89 | 96 | 58 | 84 | 40 | 44 | 68 | 52 | 6 | 58 | 27 | 48 | 82 | 62 |
| Psammothidium bioretti | 4 | 4 | 4 | 4 | 4 | 8 | 2 | 4 | 14 | 10 | 0 | 67 | 7 | 2 | 13 | 3 | 3 |
| Psammothidium helveticum | 0 | 0 | 0 | 0 | 0 | 0 | 0 | 0 | 0 | 0 | 3 | 0 | 0 | 2 | 0 | 0 | 0 |
| Psammothidium levanderi | 0 | 0 | 0 | 0 | 0 | 1 | 0 | 0 | 0 | 0 | 1 | 0 | 0 | 6 | 0 | 0 | 0 |
| Psammothidium marginulatum | 0 | 0 | 0 | 0 | 0 | 0 | 0 | 0 | 0 | 0 | 3 | 0 | 0 | 1 | 0 | 0 | 0 |
| Psammothidium rossii | 0 | 0 | 0 | 0 | 0 | 0 | 0 | 0 | 0 | 0 | 0 | 0 | 0 | 1 | 0 | 0 | 0 |
| Psammothidium subatomoides | 0 | 0 | 0 | 0 | 0 | 0 | 0 | 0 | 0 | 0 | 7 | 2 | 0 | 3 | 0 | 0 | 0 |
| Pseudostaurosira brevistriata | 4 | 0 | 0 | 0 | 2 | 10 | 0 | 0 | 0 | 0 | 0 | 0 | 0 | 0 | 0 | 0 | 0 |
| Reimeria sinuata | 2 | 0 | 0 | 2 | 0 | 2 | 0 | 0 | 7 | 2 | 0 | 32 | 0 | 0 | 2 | 2 | 0 |
| Rhopalodia sp. | 0 | 0 | 0 | 0 | 0 | 2 | 0 | 0 | 0 | 0 | 0 | 0 | 0 | 0 | 0 | 0 | 0 |
| Sellaphora bacillum | 0 | 0 | 0 | 0 | 0 | 2 | 0 | 0 | 0 | 0 | 0 | 0 | 0 | 0 | 0 | 0 | 0 |
| Sellaphora cf. pseudopupula | 0 | 0 | 0 | 0 | 0 | 0 | 0 | 0 | 2 | 0 | 0 | 0 | 0 | 0 | 0 | 0 | 0 |
| Sellaphora pupula | 2 | 2 | 0 | 0 | 0 | 0 | 2 | 0 | 0 | 0 | 0 | 0 | 0 | 0 | 0 | 0 | 4 |
| Stauroneis anceps | 0 | 0 | 0 | 0 | 0 | 0 | 0 | 2 | 2 | 0 | 0 | 0 | 0 | 0 | 2 | 0 | 0 |
| Stauroneis phoenicenteron | 0 | 0 | 0 | 2 | 0 | 0 | 0 | 0 | 0 | 0 | 0 | 0 | 0 | 0 | 0 | 0 | 0 |
| Staurosira construens | 0 | 0 | 2 | 0 | 2 | 6 | 0 | 0 | 0 | 0 | 0 | 0 | 0 | 0 | 0 | 0 | 0 |
| Staurosira venter | 0 | 0 | 0 | 0 | 47 | 0 | 0 | 0 | 0 | 0 | 0 | 0 | 0 | 0 | 0 | 0 | 0 |
| Surirella turp. sp. | 0 | 0 | 0 | 2 | 0 | 0 | 0 | 0 | 0 | 0 | 0 | 0 | 0 | 0 | 0 | 0 | 0 |
| Tabellaria fenestrata | 2 | 6 | 0 | 6 | 14 | 11 | 4 | 14 | 6 | 17 | 2 | 0 | 1 | 1 | 5 | 3 | 12 |
| Tabellaria flocculosa | 4 | 8 | 10 | 12 | 18 | 8 | 18 | 23 | 4 | 8 | 46 | 0 | 7 | 35 | 18 | 7 | 12 |
| Tetracyclus glans | 0 | 0 | 0 | 0 | 0 | 0 | 0 | 0 | 0 | 4 | 0 | 0 | 0 | 0 | 2 | 0 | 0 |
| **SUM of counts** | **347** | **367** | **347** | **359** | **344** | **350** | **343** | **428** | **348** | **372** | **334** | **304** | **377** | **341** | **450** | **365** | **360** |
