# Supplementary material for: Lake-depth related pattern of genetic and morphological diatom diversity in boreal Lake Bolshoe Toko, Eastern Siberia
Source: PLoS One. 2020 Apr 15;15(4):e0230284. doi: 10.1371/journal.pone.0230284 (PMC7159240; doi:10.1371/journal.pone.0230284)
Supplement: S4 Table — Total read counts of genetically identified diatoms in the 17 intra-lakes sites of Lake Bolshoe Toko. (DOCX) [file pone.0230284.s006.docx]

**Table S4** Total read counts of genetically identified diatoms in the 17 intra-lakes sites of Lake Bolshoe Toko.

|  |  |  | |  |  |  |  |  |  |  |  |  |  |  |  |  |  |  |
| --- | --- | --- | --- | --- | --- | --- | --- | --- | --- | --- | --- | --- | --- | --- | --- | --- | --- | --- |
|  |  | **Lake sites** | | | | | | | | | | | | | | | | |
| **Taxon** | **Short name** | **PG**  **2113.1** | **PG**  **2115.1** | **PG**  **2117.1** | **PG**  **2118.1** | **PG**  **2122.1** | **PG**  **2123.1** | **PG**  **2124** | **PG**  **2125** | **PG**  **2137.1** | **PG**  **2140.1** | **PG**  **2141.1** | **PG**  **2142.1** | **PG**  **2144.1** | **PG**  **2146.1** | **PG**  **2147.1** | **PG**  **2205.2** | **PG**  **2209.1** |
| Achnanthidae | ach_1 | 0 | 0 | 0 | 1 | 0 | 0 | 0 | 0 | 0 | 1 | 4 | 161 | 0 | 2 | 0 | 2 | 0 |
| Amphora | Amp_1 | 0 | 0 | 5 | 0 | 0 | 0 | 0 | 0 | 0 | 0 | 0 | 1 | 0 | 0 | 0 | 0 | 0 |
| Amphora | Amp_2 | 0 | 0 | 3 | 0 | 0 | 0 | 0 | 0 | 0 | 0 | 0 | 7 | 0 | 0 | 0 | 0 | 0 |
| Amphora | Amp_3 | 0 | 0 | 7 | 0 | 0 | 0 | 0 | 0 | 0 | 0 | 0 | 3 | 0 | 0 | 0 | 0 | 0 |
| Amphora | Amp_4 | 0 | 0 | 8 | 0 | 0 | 0 | 0 | 0 | 0 | 0 | 0 | 0 | 0 | 0 | 0 | 0 | 0 |
| Amphora | Amp_5 | 0 | 0 | 0 | 0 | 4 | 0 | 0 | 0 | 0 | 0 | 0 | 497 | 0 | 0 | 0 | 0 | 0 |
| Amphora | Amp_6 | 0 | 0 | 4 | 0 | 0 | 0 | 0 | 0 | 0 | 0 | 1 | 1 | 0 | 0 | 0 | 0 | 0 |
| Amphora | Amp_7 | 0 | 0 | 258 | 0 | 5 | 1 | 0 | 1 | 0 | 1 | 0 | 482 | 30 | 0 | 0 | 0 | 0 |
| Asterionella | Ast_1 | 0 | 40 | 0 | 54 | 1 | 4 | 0 | 26 | 2 | 6 | 4 | 4 | 81 | 18 | 3 | 2 | 65 |
| Aulacoseira | Aul_1 | 0 | 0 | 2 | 0 | 0 | 0 | 0 | 0 | 0 | 0 | 0 | 0 | 0 | 0 | 0 | 0 | 0 |
| Aulacoseira | Aul_10 | 0 | 0 | 12 | 0 | 0 | 0 | 3 | 7 | 0 | 0 | 0 | 0 | 0 | 0 | 0 | 0 | 0 |
| Aulacoseira | Aul_11 | 0 | 4 | 71 | 0 | 0 | 0 | 5 | 36 | 0 | 0 | 1 | 0 | 1 | 1 | 1 | 2 | 0 |
| Aulacoseira | Aul_12 | 0 | 2 | 87 | 2 | 0 | 0 | 1 | 10 | 0 | 0 | 2 | 0 | 0 | 1 | 0 | 1 | 0 |
| Aulacoseira | Aul_13 | 0 | 0 | 2 | 0 | 0 | 0 | 0 | 0 | 0 | 0 | 0 | 0 | 0 | 0 | 0 | 0 | 0 |
| Aulacoseira | Aul_14 | 0 | 0 | 2 | 0 | 0 | 0 | 0 | 8 | 0 | 0 | 0 | 0 | 0 | 0 | 0 | 0 | 0 |
| Aulacoseira | Aul_15 | 0 | 0 | 5 | 0 | 0 | 0 | 2 | 3 | 0 | 0 | 0 | 0 | 0 | 0 | 0 | 0 | 0 |
| Aulacoseira | Aul_16 | 3 | 0 | 0 | 0 | 2 | 0 | 7 | 1 | 0 | 0 | 0 | 0 | 0 | 0 | 0 | 0 | 0 |
| Aulacoseira | Aul_17 | 0 | 0 | 6 | 0 | 0 | 0 | 0 | 9 | 0 | 0 | 0 | 0 | 0 | 0 | 0 | 0 | 0 |
| Aulacoseira | Aul_18 | 0 | 0 | 15 | 1 | 0 | 0 | 0 | 3 | 0 | 0 | 0 | 0 | 0 | 0 | 0 | 1 | 0 |
| Aulacoseira | Aul_19 | 0 | 0 | 32 | 0 | 1 | 4 | 0 | 11 | 0 | 0 | 0 | 0 | 0 | 0 | 0 | 4 | 0 |
| Aulacoseira | Aul_2 | 0 | 0 | 10 | 0 | 0 | 0 | 0 | 1 | 0 | 0 | 0 | 0 | 0 | 0 | 0 | 0 | 0 |
| Aulacoseira | Aul_20 | 0 | 0 | 2 | 0 | 0 | 0 | 1 | 2 | 0 | 0 | 0 | 0 | 0 | 0 | 0 | 0 | 0 |
| Aulacoseira | Aul_21 | 0 | 1 | 0 | 0 | 0 | 0 | 1 | 10 | 0 | 0 | 1 | 0 | 0 | 0 | 0 | 0 | 0 |
| Aulacoseira | Aul_22 | 1 | 0 | 2 | 0 | 0 | 0 | 0 | 6 | 0 | 0 | 0 | 0 | 1 | 0 | 0 | 0 | 0 |
| Aulacoseira | Aul_23 | 0 | 0 | 4 | 0 | 0 | 0 | 0 | 10 | 0 | 0 | 1 | 0 | 0 | 0 | 0 | 1 | 0 |
| Aulacoseira | Aul_24 | 0 | 0 | 8 | 1 | 0 | 0 | 0 | 7 | 0 | 0 | 0 | 0 | 0 | 0 | 0 | 0 | 0 |
| Aulacoseira | Aul_25 | 0 | 6 | 0 | 17 | 30 | 5 | 19 | 3 | 0 | 0 | 0 | 0 | 0 | 2 | 2 | 0 | 0 |
| Aulacoseira | Aul_26 | 0 | 39 | 16 | 11 | 0 | 0 | 0 | 0 | 0 | 0 | 0 | 0 | 20 | 2 | 1 | 9 | 0 |
| Aulacoseira | Aul_27 | 0 | 44 | 702 | 4 | 24 | 4 | 203 | 101 | 0 | 0 | 10 | 0 | 20 | 5 | 9 | 19 | 3 |
| Aulacoseira | Aul_28 | 0 | 0 | 3 | 0 | 0 | 0 | 0 | 5 | 0 | 0 | 0 | 0 | 0 | 0 | 0 | 0 | 0 |
| Aulacoseira | Aul_29 | 0 | 0 | 4 | 1 | 0 | 0 | 0 | 1 | 0 | 0 | 0 | 0 | 1 | 0 | 0 | 0 | 0 |
| Aulacoseira | Aul_3 | 0 | 0 | 25 | 0 | 0 | 0 | 1 | 1 | 0 | 0 | 0 | 0 | 0 | 0 | 0 | 0 | 0 |
| Aulacoseira | Aul_30 | 1 | 0 | 0 | 0 | 0 | 0 | 2 | 0 | 0 | 0 | 0 | 0 | 0 | 0 | 0 | 0 | 1 |
| Aulacoseira | Aul_31 | 1 | 1 | 0 | 0 | 0 | 0 | 3 | 4 | 1 | 0 | 4 | 0 | 0 | 0 | 0 | 0 | 1 |
| Aulacoseira | Aul_32 | 4 | 2 | 45 | 0 | 1 | 1 | 1 | 11 | 0 | 0 | 1 | 1 | 4 | 0 | 0 | 1 | 0 |
| Aulacoseira | Aul_33 | 0 | 16 | 1010 | 25 | 42 | 12 | 59 | 26 | 0 | 0 | 10 | 30 | 22 | 7 | 3 | 18 | 0 |
| Aulacoseira | Aul_34 | 0 | 0 | 0 | 0 | 1 | 0 | 3 | 0 | 0 | 0 | 0 | 0 | 0 | 0 | 0 | 0 | 0 |
| Aulacoseira | Aul_35 | 0 | 0 | 0 | 0 | 11 | 0 | 5 | 1 | 0 | 0 | 1 | 0 | 0 | 0 | 1 | 0 | 1 |
| Aulacoseira | Aul_36 | 7 | 1 | 0 | 0 | 0 | 0 | 0 | 1 | 0 | 0 | 0 | 0 | 0 | 0 | 0 | 0 | 0 |
| Aulacoseira | Aul_37 | 1 | 0 | 1 | 0 | 2 | 0 | 8 | 2 | 0 | 0 | 1 | 0 | 0 | 0 | 0 | 0 | 0 |
| Aulacoseira | Aul_38 | 0 | 1 | 3 | 1 | 0 | 0 | 0 | 4 | 0 | 0 | 0 | 0 | 1 | 0 | 0 | 0 | 0 |
| Aulacoseira | Aul_39 | 0 | 11 | 1 | 4 | 0 | 0 | 0 | 2 | 0 | 0 | 2 | 0 | 8 | 1 | 0 | 5 | 0 |
| Aulacoseira | Aul_4 | 0 | 0 | 0 | 0 | 0 | 0 | 0 | 0 | 0 | 0 | 0 | 0 | 0 | 0 | 0 | 0 | 0 |
| Aulacoseira | Aul_40 | 0 | 0 | 0 | 0 | 10 | 0 | 4 | 1 | 0 | 0 | 0 | 0 | 0 | 0 | 0 | 0 | 0 |
| Aulacoseira | Aul_41 | 2 | 1 | 0 | 0 | 1 | 0 | 9 | 0 | 0 | 0 | 1 | 0 | 0 | 0 | 0 | 0 | 2 |
| Aulacoseira | Aul_42 | 12 | 0 | 0 | 0 | 0 | 0 | 0 | 0 | 0 | 0 | 0 | 0 | 8 | 0 | 0 | 0 | 0 |
| Aulacoseira | Aul_43 | 4 | 0 | 0 | 1 | 1 | 0 | 23 | 5 | 0 | 0 | 1 | 0 | 0 | 0 | 0 | 0 | 0 |
| Aulacoseira | Aul_44 | 4 | 1 | 1 | 1 | 11 | 1 | 14 | 3 | 0 | 0 | 2 | 0 | 0 | 0 | 0 | 0 | 1 |
| Aulacoseira | Aul_45 | 1 | 0 | 0 | 0 | 0 | 0 | 5 | 3 | 1 | 0 | 3 | 0 | 0 | 0 | 2 | 1 | 3 |
| Aulacoseira | Aul_46 | 5 | 1 | 1 | 2 | 3 | 0 | 22 | 5 | 0 | 0 | 2 | 0 | 0 | 0 | 0 | 0 | 0 |
| Aulacoseira | Aul_47 | 12 | 0 | 1 | 0 | 2 | 0 | 32 | 9 | 0 | 0 | 2 | 0 | 0 | 1 | 0 | 1 | 0 |
| Aulacoseira | Aul_48 | 8 | 1 | 71 | 2 | 1 | 0 | 4 | 15 | 0 | 0 | 3 | 0 | 2 | 0 | 0 | 2 | 1 |
| Aulacoseira | Aul_49 | 2 | 1 | 0 | 0 | 2 | 1 | 5 | 0 | 0 | 0 | 2 | 0 | 0 | 0 | 0 | 0 | 1 |
| Aulacoseira | Aul_5 | 0 | 0 | 6 | 0 | 0 | 0 | 0 | 0 | 0 | 0 | 0 | 0 | 0 | 0 | 0 | 0 | 0 |
| Aulacoseira | Aul_50 | 0 | 0 | 1 | 2 | 0 | 0 | 3 | 1 | 0 | 0 | 2 | 0 | 0 | 0 | 1 | 1 | 1 |
| Aulacoseira | Aul_51 | 3 | 2 | 6 | 1 | 0 | 0 | 2 | 11 | 0 | 0 | 1 | 0 | 4 | 1 | 0 | 1 | 0 |
| Aulacoseira | Aul_52 | 1 | 4 | 33 | 3 | 0 | 0 | 3 | 22 | 0 | 1 | 3 | 0 | 1 | 1 | 1 | 1 | 0 |
| Aulacoseira | Aul_53 | 0 | 178 | 18 | 177 | 10 | 3 | 0 | 12 | 0 | 4 | 31 | 8 | 903 | 40 | 10 | 133 | 0 |
| Aulacoseira | Aul_54 | 1 | 1 | 0 | 1 | 2 | 1 | 1 | 0 | 0 | 0 | 2 | 0 | 0 | 1 | 1 | 1 | 2 |
| Aulacoseira | Aul_55 | 1 | 2 | 2 | 0 | 0 | 0 | 1 | 2 | 0 | 0 | 1 | 0 | 1 | 1 | 0 | 2 | 1 |
| Aulacoseira | Aul_56 | 2 | 2 | 0 | 0 | 0 | 0 | 6 | 3 | 0 | 0 | 4 | 0 | 0 | 2 | 1 | 1 | 4 |
| Aulacoseira | Aul_57 | 0 | 1 | 0 | 1 | 0 | 0 | 0 | 3 | 1 | 2 | 3 | 0 | 2 | 3 | 3 | 1 | 0 |
| Aulacoseira | Aul_58 | 4 | 3 | 0 | 1 | 0 | 0 | 9 | 5 | 1 | 1 | 13 | 0 | 0 | 1 | 3 | 2 | 5 |
| Aulacoseira | Aul_59 | 1 | 0 | 0 | 0 | 80 | 4 | 5 | 0 | 0 | 0 | 0 | 1 | 0 | 0 | 1 | 0 | 1 |
| Aulacoseira | Aul_6 | 0 | 0 | 12 | 0 | 0 | 0 | 65 | 8 | 0 | 0 | 0 | 0 | 0 | 0 | 0 | 0 | 0 |
| Aulacoseira | Aul_60 | 10 | 15 | 4 | 11 | 0 | 0 | 6 | 65 | 2 | 3 | 9 | 0 | 7 | 4 | 3 | 5 | 0 |
| Aulacoseira | Aul_61 | 1 | 2 | 0 | 2 | 0 | 0 | 1 | 1 | 3 | 3 | 3 | 0 | 1 | 1 | 3 | 2 | 1 |
| Aulacoseira | Aul_62 | 0 | 2 | 4 | 1 | 0 | 0 | 0 | 5 | 0 | 0 | 1 | 0 | 1 | 1 | 1 | 1 | 1 |
| Aulacoseira | Aul_63 | 2 | 0 | 2 | 0 | 3 | 1 | 12 | 4 | 0 | 0 | 4 | 0 | 0 | 0 | 0 | 1 | 1 |
| Aulacoseira | Aul_64 | 0 | 4 | 8 | 2 | 0 | 0 | 1 | 9 | 0 | 0 | 4 | 0 | 3 | 2 | 1 | 1 | 1 |
| Aulacoseira | Aul_65 | 5 | 3 | 4 | 2 | 0 | 0 | 2 | 23 | 1 | 1 | 5 | 0 | 1 | 2 | 1 | 2 | 0 |
| Aulacoseira | Aul_66 | 3 | 6 | 3 | 6 | 0 | 0 | 4 | 28 | 1 | 1 | 3 | 0 | 2 | 4 | 2 | 3 | 0 |
| Aulacoseira | Aul_67 | 4 | 10 | 8 | 8 | 0 | 0 | 5 | 31 | 3 | 2 | 10 | 1 | 7 | 4 | 4 | 8 | 3 |
| Aulacoseira | Aul_68 | 5 | 13 | 10 | 11 | 0 | 0 | 7 | 44 | 1 | 2 | 11 | 0 | 2 | 6 | 3 | 8 | 1 |
| Aulacoseira | Aul_69 | 14 | 11 | 13 | 6 | 0 | 0 | 7 | 55 | 2 | 2 | 12 | 1 | 4 | 6 | 3 | 6 | 1 |
| Aulacoseira | Aul_7 | 0 | 0 | 2 | 0 | 0 | 0 | 0 | 3 | 0 | 0 | 0 | 0 | 0 | 0 | 0 | 0 | 0 |
| Aulacoseira | Aul_70 | 534 | 163 | 36 | 240 | 3 | 5 | 315 | 224 | 23 | 48 | 76 | 0 | 460 | 55 | 52 | 124 | 0 |
| Aulacoseira | Aul_71 | 620 | 123 | 7 | 81 | 682 | 173 | 1974 | 94 | 44 | 28 | 320 | 0 | 12 | 74 | 111 | 102 | 579 |
| Aulacoseira | Aul_72 | 1 | 1 | 1 | 0 | 1 | 0 | 0 | 1 | 0 | 0 | 0 | 0 | 1 | 1 | 0 | 0 | 0 |
| Aulacoseira | Aul_73 | 1 | 1 | 0 | 1 | 1 | 0 | 6 | 2 | 0 | 0 | 3 | 0 | 0 | 1 | 0 | 2 | 3 |
| Aulacoseira | Aul_74 | 4 | 2 | 0 | 1 | 1 | 0 | 12 | 3 | 1 | 0 | 10 | 0 | 0 | 1 | 2 | 2 | 3 |
| Aulacoseira | Aul_75 | 10 | 1 | 3 | 1 | 6 | 1 | 33 | 11 | 1 | 0 | 8 | 0 | 1 | 1 | 1 | 1 | 3 |
| Aulacoseira | Aul_76 | 135 | 112 | 204 | 87 | 9 | 35 | 84 | 77 | 16 | 8 | 74 | 7 | 463 | 58 | 29 | 96 | 93 |
| Aulacoseira | Aul_77 | 405 | 115 | 104 | 197 | 6 | 7 | 208 | 133 | 9 | 46 | 83 | 0 | 78 | 52 | 36 | 151 | 43 |
| Aulacoseira | Aul_78 | 990 | 94 | 117 | 120 | 27 | 13 | 373 | 182 | 24 | 31 | 78 | 1 | 129 | 54 | 45 | 116 | 52 |
| Aulacoseira | Aul_8 | 0 | 0 | 10 | 0 | 0 | 0 | 0 | 0 | 0 | 0 | 0 | 0 | 0 | 0 | 0 | 0 | 0 |
| Aulacoseira | Aul_9 | 0 | 1 | 9 | 0 | 0 | 0 | 1 | 7 | 0 | 0 | 0 | 0 | 0 | 0 | 0 | 0 | 0 |
| Chaetoceros | Cha_1 | 3 | 1 | 7 | 0 | 1 | 0 | 2 | 8 | 0 | 0 | 1 | 0 | 1 | 0 | 0 | 1 | 1 |
| Cymbellaceae | cmy_1 | 0 | 0 | 0 | 0 | 0 | 0 | 0 | 0 | 0 | 0 | 1 | 0 | 0 | 0 | 0 | 0 | 0 |
| Cymbellaceae | cmy_2 | 0 | 0 | 0 | 0 | 0 | 0 | 0 | 0 | 0 | 0 | 3 | 78 | 0 | 0 | 0 | 3 | 0 |
| Cymbellaceae | cmy_3 | 0 | 0 | 0 | 0 | 0 | 1 | 0 | 0 | 0 | 1 | 0 | 35 | 32 | 2 | 0 | 0 | 0 |
| Cymbellaceae | cmy_4 | 0 | 0 | 0 | 0 | 0 | 13 | 0 | 0 | 1 | 0 | 7 | 1805 | 0 | 1 | 0 | 0 | 0 |
| Encynema | Enc_1 | 0 | 0 | 0 | 0 | 0 | 1 | 0 | 0 | 0 | 0 | 0 | 69 | 0 | 0 | 0 | 0 | 0 |
| Fragilaria | Fra_1 | 0 | 3 | 14 | 3 | 0 | 0 | 0 | 0 | 0 | 0 | 0 | 52 | 0 | 0 | 0 | 1 | 0 |
| Fragilaria | Fra_10 | 0 | 8 | 38 | 10 | 59 | 8 | 0 | 22 | 1 | 0 | 7 | 0 | 4 | 1 | 1 | 5 | 4 |
| Fragilaria | Fra_11 | 0 | 79 | 0 | 145 | 7 | 22 | 16 | 43 | 12 | 1 | 105 | 27 | 17 | 1 | 3 | 81 | 0 |
| Fragilaria | Fra_12 | 0 | 2 | 4 | 4 | 16 | 3 | 0 | 6 | 0 | 0 | 7 | 2 | 1 | 1 | 0 | 2 | 0 |
| Fragilaria | Fra_13 | 0 | 1 | 1 | 6 | 26 | 15 | 0 | 3 | 0 | 2 | 11 | 0 | 27 | 0 | 2 | 10 | 0 |
| Fragilaria | Fra_14 | 0 | 53 | 107 | 56 | 30 | 28 | 2 | 16 | 4 | 6 | 85 | 5 | 46 | 12 | 2 | 58 | 38 |
| Fragilaria | Fra_15 | 4 | 156 | 122 | 268 | 465 | 295 | 3 | 103 | 64 | 29 | 61 | 21 | 190 | 62 | 35 | 154 | 486 |
| Fragilaria | Fra_16 | 9 | 29 | 42 | 23 | 121 | 45 | 8 | 83 | 5 | 6 | 51 | 11 | 42 | 8 | 4 | 46 | 91 |
| Fragilaria | Fra_2 | 0 | 0 | 0 | 0 | 13 | 1 | 0 | 0 | 0 | 0 | 1 | 0 | 0 | 0 | 0 | 0 | 0 |
| Fragilaria | Fra_3 | 0 | 0 | 1 | 0 | 52 | 86 | 12 | 8 | 0 | 0 | 0 | 0 | 0 | 0 | 0 | 0 | 1 |
| Fragilaria | Fra_4 | 0 | 6 | 0 | 0 | 9 | 2 | 0 | 0 | 0 | 0 | 6 | 0 | 2 | 0 | 0 | 0 | 0 |
| Fragilaria | Fra_5 | 0 | 0 | 3 | 1 | 4 | 2 | 0 | 1 | 0 | 0 | 0 | 0 | 0 | 0 | 0 | 0 | 0 |
| Fragilaria | Fra_6 | 0 | 1 | 1 | 1 | 83 | 10 | 0 | 1 | 0 | 0 | 0 | 0 | 1 | 0 | 0 | 0 | 3 |
| Fragilaria | Fra_7 | 0 | 1 | 4 | 0 | 36 | 10 | 0 | 3 | 0 | 0 | 1 | 0 | 0 | 0 | 0 | 0 | 1 |
| Fragilaria | Fra_8 | 0 | 14 | 3 | 23 | 340 | 279 | 1 | 2 | 0 | 4 | 6 | 4 | 1 | 4 | 2 | 5 | 54 |
| Fragilaria | Fra_9 | 0 | 14 | 0 | 15 | 1 | 2 | 0 | 5 | 1 | 2 | 25 | 4 | 16 | 1 | 1 | 24 | 0 |
| Fragilariaceae | fra_1 | 0 | 0 | 0 | 0 | 81 | 86 | 0 | 0 | 0 | 0 | 0 | 0 | 0 | 0 | 0 | 0 | 0 |
| Fragilariaceae | fra_10 | 0 | 1 | 2 | 1 | 6 | 0 | 0 | 0 | 0 | 0 | 0 | 0 | 0 | 0 | 0 | 0 | 0 |
| Fragilariaceae | fra_11 | 2 | 1 | 2 | 1 | 1 | 0 | 0 | 1 | 0 | 0 | 1 | 0 | 0 | 0 | 0 | 0 | 0 |
| Fragilariaceae | fra_12 | 0 | 1 | 2 | 1 | 10 | 2 | 0 | 0 | 0 | 0 | 1 | 0 | 1 | 0 | 0 | 1 | 0 |
| Fragilariaceae | fra_13 | 0 | 5 | 0 | 27 | 30 | 76 | 0 | 0 | 6 | 0 | 0 | 0 | 0 | 17 | 0 | 2 | 0 |
| Fragilariaceae | fra_14 | 0 | 0 | 3 | 1 | 11 | 2 | 0 | 2 | 0 | 0 | 0 | 0 | 0 | 0 | 0 | 0 | 0 |
| Fragilariaceae | fra_15 | 0 | 1 | 3 | 1 | 25 | 3 | 0 | 0 | 0 | 0 | 0 | 0 | 0 | 1 | 0 | 1 | 0 |
| Fragilariaceae | fra_16 | 0 | 0 | 0 | 0 | 4 | 7 | 0 | 3 | 0 | 0 | 0 | 0 | 0 | 0 | 0 | 1 | 0 |
| Fragilariaceae | fra_17 | 0 | 7 | 0 | 0 | 16 | 3 | 0 | 0 | 0 | 0 | 3 | 0 | 2 | 0 | 0 | 1 | 0 |
| Fragilariaceae | fra_18 | 4 | 0 | 4 | 0 | 1 | 0 | 3 | 12 | 0 | 0 | 1 | 0 | 0 | 0 | 0 | 0 | 0 |
| Fragilariaceae | fra_19 | 0 | 2 | 38 | 3 | 11 | 2 | 1 | 17 | 1 | 0 | 5 | 0 | 0 | 0 | 0 | 2 | 1 |
| Fragilariaceae | fra_2 | 0 | 0 | 33 | 0 | 0 | 0 | 16 | 2 | 0 | 0 | 0 | 0 | 0 | 0 | 0 | 0 | 0 |
| Fragilariaceae | fra_20 | 1 | 1 | 1 | 1 | 0 | 0 | 0 | 3 | 0 | 0 | 1 | 0 | 2 | 0 | 0 | 0 | 0 |
| Fragilariaceae | fra_21 | 2 | 0 | 6 | 1 | 2 | 1 | 0 | 4 | 0 | 0 | 1 | 0 | 1 | 0 | 0 | 1 | 0 |
| Fragilariaceae | fra_22 | 0 | 110 | 0 | 19 | 35 | 42 | 0 | 0 | 3 | 0 | 69 | 21 | 61 | 6 | 2 | 27 | 0 |
| Fragilariaceae | fra_23 | 0 | 0 | 0 | 2 | 1 | 0 | 0 | 5 | 0 | 0 | 2 | 1 | 0 | 0 | 0 | 2 | 0 |
| Fragilariaceae | fra_24 | 0 | 3 | 0 | 11 | 6 | 36 | 0 | 6 | 13 | 3 | 14 | 0 | 61 | 11 | 9 | 26 | 0 |
| Fragilariaceae | fra_25 | 0 | 2 | 0 | 2 | 1 | 0 | 1 | 5 | 0 | 0 | 1 | 0 | 0 | 0 | 0 | 0 | 0 |
| Fragilariaceae | fra_26 | 0 | 1 | 0 | 2 | 0 | 0 | 2 | 12 | 0 | 0 | 2 | 0 | 0 | 0 | 0 | 1 | 0 |
| Fragilariaceae | fra_27 | 2 | 3 | 3 | 6 | 4 | 2 | 1 | 9 | 2 | 2 | 3 | 0 | 2 | 4 | 1 | 2 | 7 |
| Fragilariaceae | fra_3 | 0 | 0 | 4 | 0 | 0 | 0 | 0 | 0 | 0 | 0 | 0 | 0 | 0 | 0 | 0 | 0 | 0 |
| Fragilariaceae | fra_4 | 0 | 0 | 2 | 0 | 1 | 0 | 0 | 1 | 0 | 0 | 0 | 0 | 0 | 0 | 0 | 0 | 0 |
| Fragilariaceae | fra_5 | 0 | 0 | 0 | 0 | 39 | 27 | 0 | 0 | 0 | 0 | 0 | 0 | 0 | 0 | 0 | 0 | 0 |
| Fragilariaceae | fra_6 | 0 | 0 | 2 | 0 | 0 | 0 | 1 | 2 | 0 | 0 | 0 | 0 | 0 | 0 | 0 | 0 | 0 |
| Fragilariaceae | fra_7 | 0 | 0 | 0 | 0 | 5 | 4 | 2 | 8 | 0 | 0 | 0 | 0 | 0 | 0 | 0 | 1 | 0 |
| Fragilariaceae | fra_8 | 0 | 11 | 1 | 11 | 50 | 35 | 0 | 0 | 0 | 0 | 18 | 0 | 0 | 0 | 3 | 0 | 0 |
| Fragilariaceae | fra_9 | 0 | 0 | 0 | 0 | 19 | 29 | 0 | 0 | 0 | 0 | 1 | 0 | 0 | 1 | 0 | 11 | 0 |
| Gomphonema | Gom_1 | 0 | 12 | 7 | 15 | 0 | 0 | 0 | 1 | 0 | 1 | 13 | 79 | 15 | 4 | 3 | 12 | 0 |
| Gophonemaceae | gom_1 | 0 | 0 | 0 | 0 | 0 | 2 | 0 | 0 | 0 | 0 | 3 | 612 | 0 | 0 | 1 | 0 | 0 |
| Lemincola | Lem_1 | 0 | 26 | 0 | 23 | 6 | 33 | 0 | 0 | 0 | 2 | 19 | 45 | 0 | 5 | 3 | 3 | 0 |
| Melosira | Mel_1 | 0 | 1 | 0 | 0 | 9 | 7 | 0 | 0 | 0 | 0 | 1 | 0 | 0 | 0 | 0 | 2 | 0 |
| Naviculaceae | nav_1 | 0 | 0 | 0 | 1 | 0 | 0 | 0 | 0 | 0 | 0 | 1 | 253 | 0 | 0 | 0 | 0 | 35 |
| Nitzschia | Nit_1 | 0 | 0 | 22 | 1 | 0 | 0 | 0 | 0 | 0 | 0 | 0 | 3 | 0 | 0 | 0 | 0 | 0 |
| Pinnularia | Pin_1 | 0 | 0 | 230 | 0 | 0 | 0 | 0 | 0 | 0 | 0 | 0 | 0 | 0 | 0 | 0 | 0 | 0 |
| Pinnularia | Pin_2 | 0 | 0 | 8 | 0 | 0 | 0 | 0 | 0 | 0 | 0 | 0 | 0 | 0 | 3 | 2 | 3 | 0 |
| Planthodium | Pla_1 | 0 | 0 | 0 | 0 | 0 | 4 | 0 | 0 | 0 | 1 | 3 | 9 | 0 | 0 | 0 | 0 | 0 |
| Sellaphora | Sel_1 | 0 | 0 | 3 | 0 | 0 | 1 | 0 | 0 | 2 | 0 | 0 | 9 | 0 | 0 | 0 | 0 | 0 |
| Sellaphora | Sel_10 | 0 | 4 | 0 | 15 | 3 | 14 | 0 | 0 | 14 | 2 | 5 | 24 | 31 | 13 | 3 | 1 | 55 |
| Sellaphora | Sel_2 | 0 | 0 | 3 | 0 | 0 | 0 | 0 | 0 | 0 | 0 | 0 | 0 | 0 | 0 | 0 | 0 | 0 |
| Sellaphora | Sel_3 | 0 | 0 | 0 | 0 | 3 | 34 | 0 | 0 | 0 | 1 | 3 | 0 | 0 | 0 | 0 | 0 | 0 |
| Sellaphora | Sel_4 | 0 | 0 | 0 | 9 | 0 | 4 | 0 | 0 | 0 | 1 | 5 | 0 | 0 | 1 | 1 | 3 | 0 |
| Sellaphora | Sel_5 | 0 | 0 | 0 | 0 | 4 | 12 | 0 | 0 | 1 | 0 | 2 | 0 | 0 | 3 | 1 | 2 | 0 |
| Sellaphora | Sel_6 | 0 | 7 | 0 | 0 | 2 | 9 | 0 | 0 | 0 | 2 | 6 | 0 | 4 | 1 | 1 | 0 | 39 |
| Sellaphora | Sel_7 | 0 | 0 | 0 | 0 | 2 | 28 | 8 | 2 | 4 | 2 | 5 | 0 | 11 | 1 | 0 | 1 | 0 |
| Sellaphora | Sel_8 | 0 | 0 | 0 | 2 | 0 | 5 | 0 | 1 | 4 | 2 | 19 | 82 | 0 | 1 | 1 | 0 | 0 |
| Sellaphora | Sel_9 | 0 | 0 | 0 | 31 | 0 | 1 | 0 | 1 | 0 | 4 | 9 | 115 | 25 | 5 | 1 | 15 | 45 |
| Stauroneis | Stu_1 | 0 | 0 | 0 | 0 | 0 | 0 | 0 | 0 | 0 | 0 | 0 | 0 | 0 | 1 | 0 | 0 | 0 |
| Stauroneis | Stu_10 | 0 | 1 | 0 | 0 | 0 | 0 | 0 | 0 | 1 | 1 | 2 | 0 | 0 | 1 | 1 | 1 | 1 |
| Stauroneis | Stu_11 | 0 | 2 | 0 | 0 | 0 | 0 | 0 | 1 | 2 | 1 | 1 | 0 | 0 | 1 | 2 | 0 | 0 |
| Stauroneis | Stu_12 | 0 | 0 | 2 | 0 | 0 | 0 | 0 | 1 | 1 | 0 | 2 | 2 | 0 | 1 | 0 | 1 | 1 |
| Stauroneis | Stu_13 | 0 | 2 | 0 | 1 | 0 | 0 | 0 | 1 | 3 | 1 | 1 | 0 | 0 | 3 | 2 | 2 | 1 |
| Stauroneis | Stu_14 | 1 | 1 | 0 | 1 | 0 | 0 | 1 | 1 | 2 | 2 | 3 | 0 | 2 | 3 | 2 | 3 | 1 |
| Stauroneis | Stu_15 | 2 | 12 | 0 | 5 | 0 | 0 | 0 | 1 | 3 | 6 | 3 | 0 | 5 | 7 | 5 | 7 | 3 |
| Stauroneis | Stu_16 | 1 | 0 | 0 | 0 | 0 | 0 | 1 | 2 | 1 | 1 | 1 | 0 | 1 | 1 | 1 | 1 | 3 |
| Stauroneis | Stu_17 | 0 | 2 | 0 | 3 | 0 | 0 | 0 | 0 | 4 | 3 | 2 | 0 | 3 | 2 | 3 | 3 | 1 |
| Stauroneis | Stu_18 | 1 | 4 | 0 | 3 | 0 | 0 | 0 | 2 | 5 | 5 | 3 | 0 | 0 | 4 | 4 | 2 | 2 |
| Stauroneis | Stu_19 | 1 | 4 | 0 | 3 | 0 | 0 | 2 | 1 | 3 | 5 | 2 | 0 | 1 | 2 | 4 | 4 | 2 |
| Stauroneis | Stu_2 | 0 | 0 | 1 | 1 | 0 | 0 | 1 | 5 | 0 | 0 | 1 | 0 | 0 | 2 | 0 | 0 | 1 |
| Stauroneis | Stu_20 | 1 | 1 | 0 | 1 | 0 | 0 | 0 | 1 | 2 | 3 | 2 | 0 | 1 | 2 | 4 | 2 | 19 |
| Stauroneis | Stu_21 | 1 | 4 | 0 | 2 | 0 | 0 | 1 | 1 | 6 | 6 | 4 | 0 | 1 | 6 | 5 | 7 | 2 |
| Stauroneis | Stu_22 | 1 | 5 | 0 | 4 | 0 | 0 | 1 | 0 | 6 | 5 | 5 | 0 | 3 | 6 | 6 | 3 | 3 |
| Stauroneis | Stu_23 | 1 | 3 | 0 | 5 | 0 | 0 | 1 | 1 | 5 | 7 | 4 | 0 | 1 | 6 | 7 | 8 | 3 |
| Stauroneis | Stu_24 | 2 | 5 | 0 | 3 | 0 | 0 | 1 | 3 | 11 | 8 | 5 | 1 | 3 | 6 | 8 | 5 | 3 |
| Stauroneis | Stu_25 | 2 | 4 | 1 | 5 | 0 | 0 | 1 | 3 | 6 | 7 | 7 | 1 | 2 | 6 | 8 | 6 | 2 |
| Stauroneis | Stu_26 | 0 | 3 | 16 | 3 | 0 | 0 | 1 | 22 | 1 | 0 | 5 | 0 | 4 | 2 | 0 | 3 | 2 |
| Stauroneis | Stu_27 | 26 | 55 | 218 | 34 | 0 | 0 | 30 | 347 | 12 | 17 | 45 | 1 | 18 | 32 | 20 | 39 | 13 |
| Stauroneis | Stu_28 | 935 | 2684 | 346 | 2345 | 3 | 1 | 545 | 1765 | 4223 | 4215 | 2746 | 89 | 1222 | 3721 | 4063 | 2887 | 1983 |
| Stauroneis | Stu_29 | 2 | 8 | 0 | 8 | 0 | 0 | 1 | 3 | 8 | 7 | 3 | 1 | 3 | 7 | 7 | 6 | 3 |
| Stauroneis | Stu_3 | 0 | 1 | 3 | 0 | 0 | 0 | 0 | 4 | 0 | 0 | 1 | 0 | 1 | 0 | 0 | 0 | 0 |
| Stauroneis | Stu_30 | 1 | 11 | 2 | 9 | 0 | 0 | 3 | 5 | 6 | 6 | 6 | 0 | 3 | 6 | 6 | 7 | 5 |
| Stauroneis | Stu_31 | 4 | 8 | 0 | 3 | 0 | 0 | 10 | 16 | 5 | 4 | 13 | 0 | 1 | 6 | 8 | 5 | 6 |
| Stauroneis | Stu_4 | 0 | 1 | 1 | 1 | 0 | 0 | 0 | 4 | 0 | 0 | 0 | 0 | 0 | 0 | 0 | 1 | 2 |
| Stauroneis | Stu_5 | 0 | 1 | 0 | 2 | 0 | 0 | 0 | 0 | 1 | 1 | 0 | 0 | 0 | 1 | 1 | 2 | 0 |
| Stauroneis | Stu_6 | 1 | 1 | 0 | 0 | 0 | 0 | 0 | 0 | 3 | 2 | 2 | 0 | 2 | 3 | 2 | 2 | 1 |
| Stauroneis | Stu_7 | 0 | 2 | 1 | 0 | 0 | 0 | 0 | 1 | 0 | 0 | 2 | 0 | 1 | 0 | 0 | 1 | 0 |
| Stauroneis | Stu_8 | 1 | 1 | 0 | 1 | 0 | 0 | 4 | 3 | 0 | 0 | 3 | 1 | 0 | 0 | 1 | 0 | 2 |
| Stauroneis | Stu_9 | 3 | 1 | 1 | 2 | 0 | 0 | 12 | 11 | 1 | 1 | 8 | 0 | 0 | 1 | 2 | 1 | 4 |
| Staurosira | Sta_1 | 0 | 0 | 0 | 0 | 11 | 19 | 0 | 0 | 0 | 0 | 0 | 0 | 0 | 0 | 0 | 0 | 0 |
| Staurosira | Sta_10 | 2 | 0 | 4 | 1 | 6 | 1 | 0 | 4 | 0 | 0 | 1 | 0 | 0 | 0 | 0 | 0 | 0 |
| Staurosira | Sta_11 | 0 | 1 | 1 | 0 | 16 | 12 | 4 | 23 | 0 | 0 | 0 | 0 | 0 | 0 | 0 | 0 | 0 |
| Staurosira | Sta_12 | 0 | 5 | 0 | 11 | 9 | 15 | 0 | 2 | 0 | 1 | 6 | 0 | 0 | 3 | 3 | 16 | 0 |
| Staurosira | Sta_13 | 0 | 1 | 0 | 0 | 112 | 317 | 6 | 0 | 1 | 0 | 1 | 0 | 16 | 1 | 0 | 6 | 0 |
| Staurosira | Sta_14 | 0 | 2 | 1 | 0 | 7 | 3 | 0 | 0 | 0 | 0 | 2 | 0 | 0 | 0 | 0 | 0 | 0 |
| Staurosira | Sta_15 | 0 | 0 | 1 | 1 | 28 | 13 | 0 | 4 | 1 | 0 | 1 | 2 | 1 | 0 | 0 | 0 | 0 |
| Staurosira | Sta_16 | 2 | 7 | 1 | 1 | 26 | 16 | 0 | 3 | 0 | 0 | 3 | 0 | 0 | 0 | 0 | 1 | 0 |
| Staurosira | Sta_17 | 0 | 34 | 0 | 0 | 56 | 65 | 0 | 1 | 2 | 0 | 4 | 2 | 1 | 0 | 2 | 5 | 0 |
| Staurosira | Sta_18 | 1 | 2 | 15 | 2 | 29 | 7 | 1 | 10 | 0 | 0 | 3 | 0 | 0 | 1 | 0 | 1 | 1 |
| Staurosira | Sta_19 | 9 | 51 | 30 | 2 | 91 | 64 | 1 | 13 | 0 | 0 | 5 | 0 | 26 | 2 | 1 | 0 | 0 |
| Staurosira | Sta_2 | 0 | 0 | 0 | 0 | 21 | 42 | 0 | 0 | 0 | 0 | 0 | 0 | 0 | 0 | 0 | 0 | 0 |
| Staurosira | Sta_20 | 0 | 21 | 15 | 18 | 83 | 388 | 20 | 62 | 0 | 8 | 7 | 0 | 0 | 4 | 6 | 21 | 0 |
| Staurosira | Sta_21 | 1 | 0 | 0 | 0 | 11 | 3 | 0 | 0 | 0 | 0 | 1 | 0 | 0 | 1 | 0 | 1 | 2 |
| Staurosira | Sta_22 | 286 | 50 | 95 | 103 | 107 | 484 | 8 | 35 | 1 | 6 | 111 | 8 | 92 | 9 | 5 | 47 | 0 |
| Staurosira | Sta_23 | 0 | 2 | 1 | 7 | 164 | 230 | 2 | 40 | 0 | 1 | 6 | 16 | 4 | 3 | 2 | 1 | 2 |
| Staurosira | Sta_24 | 0 | 50 | 4 | 181 | 166 | 253 | 7 | 56 | 12 | 8 | 112 | 9 | 219 | 41 | 17 | 112 | 0 |
| Staurosira | Sta_25 | 566 | 38 | 163 | 99 | 1070 | 1118 | 367 | 414 | 87 | 100 | 114 | 3 | 142 | 206 | 68 | 125 | 888 |
| Staurosira | Sta_3 | 0 | 0 | 0 | 0 | 12 | 7 | 0 | 0 | 0 | 0 | 0 | 0 | 0 | 0 | 0 | 0 | 0 |
| Staurosira | Sta_4 | 0 | 0 | 0 | 0 | 2 | 6 | 0 | 0 | 0 | 0 | 18 | 0 | 0 | 1 | 0 | 5 | 0 |
| Staurosira | Sta_5 | 0 | 0 | 0 | 0 | 11 | 5 | 0 | 0 | 0 | 0 | 0 | 0 | 0 | 0 | 0 | 0 | 0 |
| Staurosira | Sta_6 | 0 | 0 | 0 | 0 | 25 | 25 | 0 | 0 | 0 | 0 | 1 | 3 | 0 | 0 | 0 | 1 | 0 |
| Staurosira | Sta_7 | 0 | 0 | 0 | 2 | 0 | 1 | 0 | 4 | 0 | 0 | 3 | 0 | 0 | 0 | 0 | 0 | 0 |
| Staurosira | Sta_8 | 1 | 0 | 3 | 0 | 1 | 1 | 0 | 1 | 0 | 0 | 0 | 0 | 0 | 0 | 0 | 0 | 0 |
| Staurosira | Sta_9 | 0 | 0 | 1 | 0 | 7 | 1 | 0 | 1 | 0 | 0 | 0 | 1 | 0 | 0 | 0 | 0 | 0 |
| Tabellaria | Tab_1 | 0 | 0 | 0 | 0 | 0 | 0 | 0 | 0 | 0 | 0 | 0 | 0 | 50 | 0 | 0 | 0 | 0 |
