# Supplementary material for: Lake-depth related pattern of genetic and morphological diatom diversity in boreal Lake Bolshoe Toko, Eastern Siberia
Source: PLoS One. 2020 Apr 15;15(4):e0230284. doi: 10.1371/journal.pone.0230284 (PMC7159240; doi:10.1371/journal.pone.0230284)
Supplement: S5 Table — Compilation of total sample count, rarefied sample count, water depth, taxonomic alpha diversity (richness and effective number of species) and phylogenetic diversity (NRI-Net relatedness index, pa-presence/absence data; aw-average weighted abundance data) for the genera Aulacoseira and Staurosira of the 17 intra-lake localities in Bolshoe Toko. (DOCX) [file pone.0230284.s007.docx]

**Table S5** Compilation of total sample count, rarefied sample count, water depth, taxonomic alpha diversity (richness and Simpson diversity) and phylogenetic diversity (NRI-Net relatedness index, pa-presence/absence data; aw-average weighted abundance data) for the genera *Aulacoseira* and *Staurosira* of the 17 intra-lake localities in Bolshoe Toko.

| **lake sites** | **total sample count** | **rarefied sample count** | **water depth**  **(m)** | **Richness**  **(number unique sequence types)** | **Simpson diversity** | **richness**  **(after sample**  **rarefaction)** | **NRI (pa)** | ***P*** | **NRI**  **(aw)** | ***P*** |
| --- | --- | --- | --- | --- | --- | --- | --- | --- | --- | --- |
| ***Aulacoseira*** | | | | | | | | | | |
| PG2113.1 | 55171 | 50 | 62 | 55 | 4.36 | 41 | 3.70 | 0.002 | 1.95 | 0.031 |
| PG2115.1 | 4225 | 50 | 45.5 | 64 | 9.10 | 61 | 2.91 | 0.003 | 2.25 | 0.014 |
| PG2117.1 | 129048 | 50 | 36.9 | 71 | 4.79 | 54 | 3.93 | 0.001 | 1.57 | 0.052 |
| PG2118.1 | 3290 | 50 | 62.3 | 52 | 6.82 | 51 | 2.15 | 0.016 | 2.73 | 0.003 |
| PG2122.1 | 6888 | 50 | 18.3 | 40 | 1.99 | 37 | 1.07 | 0.139 | 1.35 | 0.099 |
| PG2123.1 | 1757 | 50 | 6.1 | 32 | 2.36 | 31 | 1.99 | 0.025 | 0.51 | 0.324 |
| PG2124 | 17208 | 50 | 30 | 74 | 3.05 | 49 | 0.35 | 0.37 | 0.27 | 0.412 |
| PG2125 | 18965 | 50 | 30 | 75 | 13.76 | 70 | 4.41 | 0.001 | 3.99 | 0.001 |
| PG2137.1 | 1276 | 50 | 5.8 | 33 | 5.68 | 33 | 2.45 | 0.008 | 1.54 | 0.062 |
| PG2140.1 | 4434 | 50 | 25 | 45 | 5.58 | 39 | 2.36 | 0.013 | 2.43 | 0.009 |
| PG2141.1 | 5414 | 50 | 27 | 64 | 5.35 | 59 | 1.21 | 0.106 | 0.97 | 0.182 |
| PG2142.1 | 50 | 50 | 0 | 8 | 2.46 | 8 | 2.18 | 0.018 | 1.18 | 0.127 |
| PG2144.1 | 6463 | 50 | 36.8 | 41 | 3.74 | 34 | 4.61 | 0.001 | 2.04 | 0.016 |
| PG2146.1 | 992 | 50 | 5 | 43 | 8.39 | 43 | 2.00 | 0.021 | 2.26 | 0.012 |
| PG2147.1 | 3241 | 50 | 11 | 54 | 5.90 | 53 | 2.60 | 0.005 | 1.24 | 0.113 |
| PG2205.2 | 4810 | 50 | 68.3 | 65 | 7.86 | 60 | 2.46 | 0.01 | 2.50 | 0.004 |
| PG2209.1 | 10205 | 50 | 31.2 | 48 | 1.91 | 41 | 0.07 | 0.471 | 0.27 | 0.417 |
| ***Staurosira*** | | | | | | | | | | |
| PG2113.1 | 17040 | 45 | 62 | 13 | 1.87 | 12 | 1.14 | 0.134 | 0.02 | 0.462 |
| PG2115.1 | 1119 | 45 | 45.5 | 17 | 6.60 | 17 | 1.24 | 0.118 | 1.10 | 0.140 |
| PG2117.1 | 15495 | 45 | 36.9 | 16 | 3.01 | 16 | 2.81 | 0.002 | 0.54 | 0.310 |
| PG2118.1 | 1367 | 45 | 62.3 | 14 | 3.40 | 14 | 0.86 | 0.209 | 0.58 | 0.290 |
| PG2122.1 | 14622 | 45 | 18.3 | 24 | 3.42 | 24 | -0.14 | 0.584 | 1.39 | 0.064 |
| PG2123.1 | 19637 | 45 | 6.1 | 25 | 5.13 | 25 | NA | 0.501 | 0.61 | 0.277 |
| PG2124 | 1989 | 45 | 30 | 13 | 1.29 | 13 | 1.30 | 0.097 | 0.72 | 0.239 |
| PG2125 | 9108 | 45 | 30 | 21 | 2.51 | 21 | 1.64 | 0.064 | 1.49 | 0.047 |
| PG2137.1 | 957 | 45 | 5.8 | 12 | 1.44 | 12 | 0.33 | 0.383 | 1.08 | 0.136 |
| PG2140.1 | 3028 | 45 | 25 | 16 | 1.56 | 16 | 1.51 | 0.076 | 0.93 | 0.161 |
| PG2141.1 | 2592 | 45 | 27 | 20 | 4.11 | 20 | 0.15 | 0.396 | 0.39 | 0.382 |
| PG2142.1 | 45 | 45 | 0 | 8 | 4.39 | 8 | 2.07 | 0.011 | 1.74 | 0.020 |
| PG2144.1 | 1500 | 45 | 36.8 | 14 | 3.26 | 14 | 1.95 | 0.023 | 0.73 | 0.236 |
| PG2146.1 | 678 | 45 | 5 | 15 | 1.67 | 15 | 0.33 | 0.381 | 1.05 | 0.145 |
| PG2147.1 | 1024 | 45 | 11 | 16 | 2.26 | 16 | 0.33 | 0.381 | 0.99 | 0.156 |
| PG2205.2 | 1976 | 45 | 68.3 | 21 | 3.80 | 21 | -0.14 | 0.522 | 0.55 | 0.315 |
| PG2209.1 | 11144 | 45 | 31.2 | 8 | 1.01 | 8 | 2.24 | 0.005 | 2.03 | 0.003 |

**Figure S1** Results of the PROCRUSTES analyses. Procrustes error plots indicate A – the distance between diatom assembly data derived from genetic and morphological data, B – residuals of the comparison between PCA site scores derived of genetic and morphological data (residuals were ordered according to water de­­­­pth of the lake, beginning from the shallowest site). Dashed and solid lines are the first, second and third quartiles.

B

A

**Figure S2** Scatter plots show the correlations between water depth and richness (a) and richness and NRI (from presence/absence (pa) data) (b) for the genus *Aulacoseira.* The plot (c) indicates the correlation between water depth and richness and (d) between richness and NRI (from presence/absence (pa) data) for the genus *Staurosira*.

a

b

c

d
